# Metabolic Regulation of Immune Responses: Molecular Mechanisms, Diseases, and Therapeutic Targets

**DOI:** 10.1002/mco2.70801

**Published:** 2026-06-08

**Authors:** Chunwei Li, Ziqiang Liu, Dezheng Kong, Zhengze Li, Yiming Yan, Yanyu Dong, Lili Zhu, JiaNing Cao, Zhirui Fan, Gautam Sethi, Lifeng Li

**Affiliations:** ^1^ National Engineering Laboratory for Internet Medical Systems and Applications The First Affiliated Hospital of Zhengzhou University Zhengzhou China; ^2^ Department of Pharmacology Henan Provincial People's Hospital Zhengzhou China; ^3^ Department of Radiation Oncology Nanfang Hospital Southern Medical University Guangzhou China; ^4^ Colorectal Cancer Center Department of General Surgery West China Hospital Sichuan University Chengdu China; ^5^ The First Affiliated Hospital of Zhengzhou University Zhengzhou China; ^6^ Zhengzhou University First Affiliated Hospital Department of Radiation Oncology Zhengzhou China; ^7^ School of Stomatology Zhengzhou University Zhengzhou China; ^8^ Department of Chinese and Western Integrative Medicine the First Affiliated Hospital of Zhengzhou University Zhengzhou China; ^9^ Department of Pharmacology and NUS Centre for Cancer Research (N2CR) School of Medicine Yong Loo Lin National University of Singapore Singapore Singapore; ^10^ Cancer Center The First Affiliated Hospital of Zhengzhou University Zhengzhou China; ^11^ Traditional Chinese Medicine Hospital of Xinjiang Uyghur Autonomous Region Urumqi China

**Keywords:** immunometabolism, tumor microenvironment, FBP1, cancer immunotherapy

## Abstract

Cancer‐associated metabolic reprogramming profoundly reshapes the tumor microenvironment (TME), emerging as a central driver of immune evasion and therapeutic resistance. Increasing evidence indicates that metabolic enzymes function not only as bioenergetic regulators but also as active modulators of immune signaling, immune cell fate, and immune checkpoint expression. To elucidate these complex immunometabolic networks, this review utilizes fructose‐1,6‐bisphosphatase 1 (FBP1)—a key gluconeogenic enzyme—as a paradigmatic metabolic gatekeeper to illustrate how metabolic dysregulation drives tumor progression. By examining both the canonical metabolic effects and noncanonical signaling mechanisms of such enzymes, we synthesize recent advances demonstrating how metabolic rewiring promotes glycolytic reprogramming, immune suppression, and resistance to immunotherapy. Specifically, we explore broad mechanisms of immune evasion, including STAT3–PD‐L1 regulation, modulation of innate immune surveillance, T cell exhaustion, and remodeling of stromal and fibrotic tumor niches. Furthermore, we discuss emerging therapeutic strategies targeting these immunometabolic pathways, encompassing small‐molecule modulators, vitamin‐ and gene‐based interventions, nanotechnology‐enabled delivery systems, and metabolism‐informed combination immunotherapy. Finally, we highlight key challenges, including metabolic heterogeneity and context‐dependent enzyme function, emphasizing the need for biomarker‐guided precision strategies to translate fundamental immunometabolic insights into durable and safe cancer therapies.

## Graphical abstract

FBP1 loss drives immune evasion and therapy resistance by enhancing glycolysis, STAT3 activation, and PD‐L1 expression, leading to T cell exhaustion and NK cell inhibition. FBP1 restoration, via LNP‐mRNA or epigenetic modulation, reverses these immunosuppressive effects, reactivates cytotoxic T cells, promotes M1 macrophage polarization, and enhances antitumor immunity

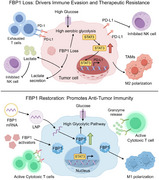

## 1 Introduction

Cancer immunotherapy has fundamentally reshaped the landscape of oncology; however, durable clinical responses are achieved in only a subset of patients. Increasing evidence indicates that metabolic reprogramming within the tumor microenvironment (TME) plays a decisive role in shaping antitumor immunity, immune escape, and therapeutic resistance. Initially viewed merely as an adaptive response to sustain rapid proliferation, tumor‐associated metabolic alterations are now recognized as active regulators of immune surveillance and inflammatory signaling. Consequently, the emerging field of immunometabolism highlights cellular metabolism as a critical interface linking oncogenic signaling, immune cell function, and treatment responsiveness.

Among metabolic pathways implicated in immune regulation, glucose metabolism occupies a central position due to its dual role in energy production and immune effector function. To fully comprehend the impact of these pathways, it is necessary to examine the specific enzymatic nodes that control them. Fructose‐1,6‐bisphosphatase 1 (FBP1), a rate‐limiting enzyme of gluconeogenesis, serves as an exemplary model for this intersection. While traditionally regarded as a metabolic tumor suppressor whose loss promotes aerobic glycolysis, recent studies reveal that enzymes like FBP1 exert broader biological effects that extend far beyond simple glucose metabolism. The dysregulation of these metabolic gatekeepers influences immune cell infiltration, immune checkpoint expression, and inflammatory signaling within the TME, firmly positioning them as important immunometabolic regulators.

Currently, existing studies on metabolic‐immune crosstalk remain fragmented, often focusing on individual tumor types or isolated mechanisms, and a comprehensive synthesis of FBP1‐centered immunometabolic regulation is still lacking. Accumulating evidence suggests that metabolic enzymes such as FBP1 are not passive downstream targets of oncogenic signaling but active determinants of immune tolerance and therapeutic sensitivity. Their functions are dynamically shaped by epigenetic regulation, posttranslational modifications, and oncogenic signaling pathways, resulting in context‐dependent effects on tumor immunity. These intrinsic regulatory mechanisms introduce an additional layer of complexity while simultaneously offering opportunities for precision targeting and biomarker‐guided therapeutic strategies. Despite growing interest, how these regulatory axes converge to influence immune responses and clinical outcomes has not been systematically reviewed.

In this review, we provide an integrated and mechanism‐oriented overview of how metabolic regulation interfaces with immune signaling in cancer, utilizing FBP1 as a primary molecular lens. We first summarize the molecular mechanisms linking metabolism to immune regulation, emphasizing metabolite‐mediated immune signaling, noncanonical immune functions of metabolic enzymes, and intrinsic regulatory mechanisms controlling enzyme activity. We then discuss tumor‐specific patterns of FBP1 dysregulation and their immunological consequences within the TME. Building on these insights, we examine emerging preclinical evidence and translational strategies, including metabolism‐informed combination immunotherapy and biomarker‐guided patient stratification. Finally, we highlight current challenges and future perspectives, aiming to delineate a conceptual framework for translating immunometabolic insights into clinically effective and safe cancer therapies.

## 2 Overview of Metabolic Regulation in Immune Responses

Immunometabolism has emerged as a central framework for understanding how metabolic programs dynamically regulate immune cell fate, function, and interactions within the TME. This section provides a systematic overview of the conceptual foundations of immunometabolism and the key metabolic pathways that shape immune responses, with a focus on the reciprocal regulatory relationship between cellular metabolism and immune function. This section first introduces the conceptual framework of immunometabolism, elaborating on the paradigm shift from viewing metabolism merely as bioenergetic support to recognizing it as an active determinant of immune signaling, transcriptional regulation, and functional polarization. Next, it systematically discusses the major metabolic pathways, including glucose metabolism, mitochondrial oxidative phosphorylation, amino acid metabolism, and lipid metabolism, illustrating how each pathway regulates immune cell activation, differentiation, and effector function under both physiological and pathological conditions. Within this section, particular attention is given to the role of key metabolic enzymes such as FBP1 as integrative nodes connecting metabolic reprogramming to immune regulation. Thus, this section establishes a theoretical basis for understanding how metabolic dysregulation contributes to immune evasion and provides a framework for the subsequent sections that explore the specific mechanisms of FBP1‐mediated metabolic‐immune crosstalk across different disease contexts.

### 2.1 Conceptual Framework of Immunometabolism

Immunometabolism refers to the dynamic and reciprocal regulation between cellular metabolic programs and immune responses. Initially regarded as a passive provider of bioenergetic support, cellular metabolism is now recognized as a central determinant of immune cell activation, differentiation, effector function, and immune tolerance. Accumulating evidence demonstrates that metabolic pathways not only sustain immune cell survival but also actively shape immune signaling, transcriptional programs, and functional fate decisions [1, 2, 3].

A defining feature of immunometabolism is metabolic reprogramming, a process by which immune and tumor cells adapt their metabolic profiles in response to environmental cues such as hypoxia, nutrient availability, inflammatory stimuli, and therapeutic stress. For example, enhanced aerobic glycolysis, long recognized as a hallmark of cancer cells (the Warburg effect), has also been observed in activated immune cells, including effector T cells and NK cells, enabling rapid ATP generation and biosynthetic precursor supply [4]. Conversely, suppression of glycolysis and a shift toward oxidative phosphorylation or gluconeogenesis‐associated pathways can profoundly alter immune cell functionality and immune surveillance [1, 2].

Beyond serving as metabolic flux regulators, key metabolic enzymes exert noncanonical regulatory roles in immune modulation. Enzymes involved in glucose metabolism, such as FBP1, have emerged as critical molecular nodes that integrate metabolic status with immune signaling pathways. Alterations in the expression, localization, or posttranslational modification of these enzymes can reshape intracellular signaling networks, transcription factor activity, and immune checkpoint regulation, thereby influencing immune escape, inflammation, and antitumor immunity [5].

**FIGURE 1:**
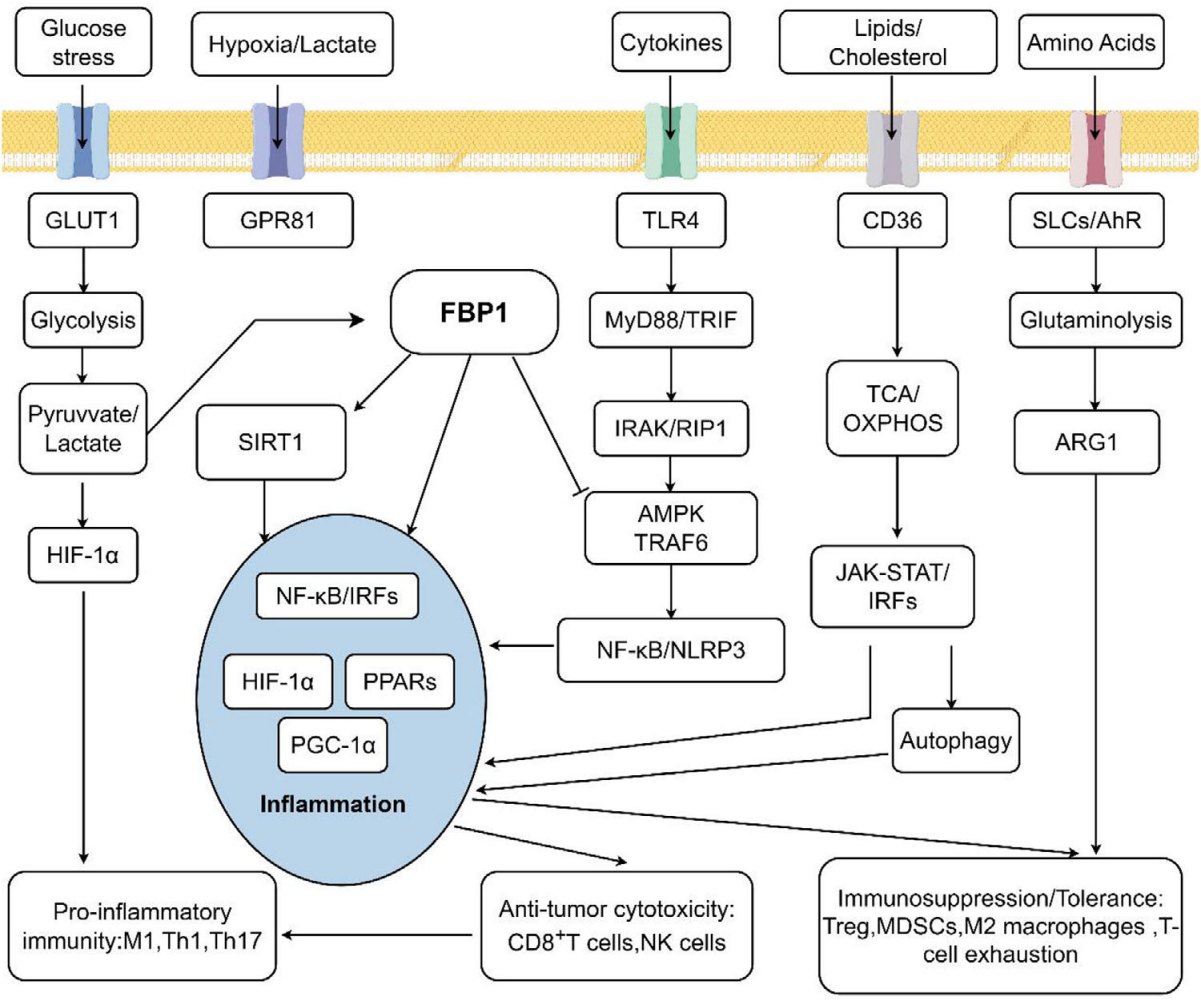
This illustration delineates the complex interplay between metabolic stressors and immune regulatory pathways within the tumor microenvironment. Diverse microenvironmental stimuli, including glucose stress, hypoxia/lactate accumulation, cytokines, cholesterol, and other lipids/amino acids, are sensed by specific receptors or transporters such as GLUT1, GPR81, TLR4, CD36, and SLCs/AhR. These upstream signals converge on key metabolic hubs, notably FBP1 and AMPK, which orchestrate a bifurcated cellular response. On one axis, alterations in glycolysis, glutaminolysis, and the TCA cycle/OXPHOS system modulate core energy metabolism, intersecting with nutrient‐sensing regulators like SIRT1, HIF‐1α, and PGC‐1α. On a parallel axis, these metabolic shifts are hardwired into immune signaling circuits involving MyD88/TRIF, IRAK/RIP1, TRAF6, and JAK‐STAT/NF‐κB/IRFs pathways. The integration of these metabolic and immune signals by transcription factors such as HIF‐1α, PPARs, and NF‐κB/NLRP3 dictates divergent functional outcomes: promoting either pro‐inflammatory, antitumor immunity (driven by M1 macrophages, Th1, Th17 cells, CD8+ T cells, and NK cells) or fostering a state of immunosuppression and tolerance (mediated by Tregs, MDSCs, M2 macrophages, and T‐cell exhaustion). GLUT1, glucose transporter 1; GPR81, G protein‐coupled receptor 81; TLR4, toll‐like receptor 4; CD36, cluster of differentiation 36; SLCs, solute carriers; AhR, aryl hydrocarbon receptor; AMPK, AMP‐activated protein kinase; TCA, tricarboxylic acid; OXPHOS, oxidative phosphorylation; SIRT1, sirtuin 1; HIF‐1α, hypoxia‐inducible factor 1‐alpha; PGC‐1α, peroxisome proliferator‐activated receptor gamma coactivator 1‐alpha; MyD88, myeloid differentiation primary response 88; TRIF, TIR‐domain‐containing adapter‐inducing interferon‐β; IRAK, interleukin‐1 receptor‐associated kinase; RIP1, receptor‐interacting protein 1; TRAF6, TNF receptor‐associated factor 6; JAK‐STAT, janus kinase‐signal transducer and activator of transcription; NF‐κB, nuclear factor kappa‐light‐chain‐enhancer of activated B cells; IRFs, interferon regulatory factors; NLRP3, NLR family pyrin domain containing 3; PPARs, peroxisome proliferator‐activated receptors; Th, T helper; Treg, regulatory T cell; MDSCs, myeloid‐derived suppressor cells; NK, natural killer.

Importantly, immunometabolic regulation operates in a highly context‐dependent manner. Intrinsic regulatory mechanisms, including epigenetic modification, phosphorylation, and ubiquitin‐mediated proteostasis, tightly control the activity and stability of metabolic enzymes, enabling cells to fine‐tune immune responses under physiological and pathological conditions [6, 7, 8]. Together, these findings establish immunometabolism as an integrated framework linking metabolic reprogramming to immune regulation, providing a conceptual foundation for understanding how metabolic dysregulation contributes to immune‐related diseases and for identifying novel therapeutic targets. Collectively, these extracellular metabolic cues and intracellular signaling cascades are integrated not through isolated pathways, but via a coordinated network of nutrient transporters, metabolic sensors, and transcriptional nodes—including AMPK, HIF‐1α, NF‐κB, and PPARs—that ultimately dictate the functional polarization of immune cells within the tumor microenvironment (Figure 1).

### 2.2 Key Metabolic Pathways Shaping Immune Cell Function

Immune cell activation, differentiation, and effector function are tightly regulated by cellular metabolic programs. In recent years, immunometabolism has emerged as a central framework explaining how immune responses are dynamically shaped by intracellular metabolic states and extracellular nutrient availability. Rather than serving solely as energy‐producing processes, metabolic pathways actively participate in immune signaling, fate determination, and functional polarization of immune cells.

At a systemic level, immune responses are governed by coordinated changes in multiple metabolic pathways, including glucose metabolism, mitochondrial oxidative phosphorylation, amino acid utilization, and lipid metabolism. These pathways collectively determine cellular bioenergetics, redox balance, and the availability of biosynthetic and epigenetic substrates. Importantly, immune cells exhibit distinct metabolic preferences depending on their activation state and functional role. For example, rapid effector responses are typically associated with enhanced glycolytic flux, whereas long‐lived memory and regulatory immune phenotypes rely more heavily on mitochondrial metabolism and oxidative phosphorylation. Such metabolic flexibility enables immune cells to adapt to fluctuating environmental and inflammatory conditions.

#### 2.2.1 Glucose Metabolism

Glucose metabolism is a fundamental regulator of immune cell activation and effector function within the TME. Activated cytotoxic T lymphocytes and natural killer (NK) cells rely predominantly on aerobic glycolysis to sustain rapid proliferation, cytokine production, and cytotoxic activity. However, excessive glycolytic reprogramming in tumor cells profoundly reshapes nutrient availability and metabolite composition in the TME, leading to glucose deprivation and lactate accumulation.

Loss or downregulation of FBP1 enhances the Warburg effect in tumor cells, intensifying glucose competition between malignant and immune cells [3, 5]. This metabolic imbalance indirectly suppresses antitumor immunity by impairing glycolysis‐dependent immune effector functions. Elevated lactate levels further inhibit T cell and NK cell activity, promote immune exhaustion, and facilitate immune escape [9, 10].

Importantly, restoration of FBP1 expression attenuates aberrant glycolysis and reduces PD‐L1 upregulation through suppression of STAT3‐dependent transcriptional programs, thereby alleviating glucose‐driven immune suppression and improving responsiveness to immune checkpoint blockade [5, 10, 11]. Thus, glucose metabolism functions as a critical metabolic checkpoint linking tumor metabolic reprogramming to immune regulation.

#### 2.2.2 Mitochondrial Metabolism and Oxidative Phosphorylation

Mitochondrial metabolism and oxidative phosphorylation (OXPHOS) are essential for maintaining immune cell persistence, functional stability, and long‐term antitumor surveillance. Unlike short‐lived effector responses driven primarily by glycolysis, memory T cells and sustained immune responses depend heavily on intact mitochondrial respiration and metabolic flexibility.

In the TME, tumor‐driven metabolic reprogramming disrupts mitochondrial homeostasis in immune cells, leading to impaired OXPHOS capacity and immune exhaustion. Accumulating evidence suggests that FBP1‐mediated restraint of excessive glycolysis indirectly preserves mitochondrial integrity and metabolic balance in immune cells [5, 9]. Conversely, FBP1 deficiency exacerbates metabolic stress, promotes mitochondrial dysfunction, and facilitates immune checkpoint activation.

Therefore, mitochondrial metabolism represents a key axis connecting metabolic homeostasis to immune competence, reinforcing the role of FBP1 as an integrative regulator of metabolic and immune signaling within the TME.

#### 2.2.3 Amino Acid Metabolism

Amino acid metabolism plays a pivotal role in regulating immune cell activation, differentiation, and inflammatory signaling within the TME. Metabolic competition for amino acids, together with oncogenic and inflammatory cues, reshapes immune responses by modulating transcriptional programs and signal transduction pathways.

FBP1 deficiency is closely associated with sustained activation of the STAT3 pathway and increased PD‐L1 expression, processes that are functionally intertwined with altered amino acid metabolism and cellular stress responses [12, 13, 14, 15, 16, 17]. Persistent STAT3 signaling suppresses cytotoxic T cell activity and promotes immune tolerance, thereby facilitating tumor immune escape. In addition, dysregulated amino acid metabolism contributes to macrophage polarization and reinforces immunosuppressive niches within the TME.

Overall, aberrant amino acid metabolic pathways cooperate with the deficiency of metabolic enzymes such as FBP1 to amplify immune evasion and shape immunological outcomes in cancer.

#### 2.2.4 Lipid Metabolism

Lipid metabolism is increasingly recognized as a critical determinant of immune cell polarization and function in the TME. Enhanced lipid utilization and fatty acid oxidation are characteristic features of immunosuppressive immune cell subsets, particularly M2‐like tumor‐associated macrophages (TAMs).

High FBP1 expression in macrophages constrains glycolytic activity and favors lipid‐dependent metabolic programs, thereby promoting M2 polarization and secretion of immunosuppressive cytokines such as IL‐10 and TGF‐β [9]. This metabolic phenotype supports tumor progression and attenuates antitumor immune responses. Conversely, metabolic interventions that disrupt FBP1‐mediated constraints or reprogram lipid metabolism can shift macrophages toward a pro‐inflammatory phenotype and enhance the efficacy of immune checkpoint blockade [10].

These findings underscore lipid metabolism as a downstream effector of FBP1‐regulated metabolic‐immune crosstalk and highlight its therapeutic relevance in reshaping the TME. Therapeutic modulation of key metabolic pathways can reprogram immune cell function by alleviating glucose competition, restoring mitochondrial homeostasis, fine‐tuning amino acid signaling, and reversing lipid‐dependent immunosuppression, thereby reinstating antitumor immunity (Table 1).

**TABLE 1:** Key metabolic pathways affecting immune cell function.

Metabolic pathways	Key characteristics and functions	Impact on immune cell function	Role of FBP1
Glucose metabolism	Aerobic glycolysis sustains rapid proliferation, cytokine secretion, and cytotoxic activity of T/NK cells; excessive glycolysis depletes glucose and accumulates lactate	Lactate inhibits T and NK cell activity, promoting immune exhaustion and escape [9, 10]	Inhibits glycolysis, alleviates PD‐L1‐mediated immunosuppression, and enhances immune checkpoint‐blockade response [10, 11, 18]
Mitochondrial metabolism and OXPHOS	Maintains durable immune responses (e.g., memory T cells) and long‐term antitumor surveillance	The TME disrupts mitochondrial homeostasis, leading to impaired OXPHOS and immune exhaustion	By inhibiting excessive glycolysis, it helps maintain mitochondrial integrity and metabolic balance in immune cells [9, 18]
Amino acid metabolism	Regulates immune cell activation, differentiation, and inflammatory signaling	Dysregulation promotes sustained STAT3 activation and PD‐L1 upregulation, suppressing T cell activity and fostering tolerance	Inhibits STAT3 pathway activation and PD‐L1 expression, working with amino acid metabolism to suppress immune escape [12, 13, 14, 15, 16, 17]
Lipid Metabolism	Lipid utilization and fatty acid oxidation characterize immunosuppressive subsets like M2‐like TAMs	Promotes M2 macrophage polarization and secretion of IL‐10/TGF‐β, weakening antitumor immunity [9]	Inhibits glycolysis and promotes lipid‐dependent metabolic programs, driving M2 polarization [9]

Abbreviations: FBP1, fructose‐1,6‐bisphosphatase 1; IL‐10, interleukin‐10; OXPHOS, oxidative phosphorylation; PD‐L1, programmed death‐ligand 1; STAT3, signal transducer and activator of transcription 3; TAMs, tumor‐associated macrophages; TGF‐β, transforming growth factor‐β; TME, tumor microenvironment.

In summary, the immunometabolic framework established in this chapter demonstrates that metabolic reprogramming actively shapes immune cell functions through interconnected pathways, including glucose metabolism, mitochondrial metabolism, amino acid metabolism, and lipid metabolism. Within this framework, metabolic enzymes serve as key nodes linking metabolic status to immune outcomes. FBP1 represents a typical example of such nodes, integrating its metabolic and immune regulatory functions. The following sections will build on this foundation to dissect the molecular mechanisms by which FBP1 mediates metabolic‐immune crosstalk in cancer contexts.

## 3 Molecular Mechanisms Linking Metabolism to Immune Regulation

Metabolic reprogramming not only supports tumor cell proliferation but also exerts profound regulatory effects on immune surveillance, inflammatory signaling, and immune tolerance. Increasing evidence indicates that metabolites, metabolic enzymes, and their intrinsic regulatory mechanisms collectively form a multilayered network linking cellular metabolic states to immune regulation within the TME. This section outlines the principal molecular mechanisms through which metabolism governs immune responses, with particular emphasis on metabolite‐mediated signaling, noncanonical immune functions of metabolic enzymes, and intrinsic regulatory mechanisms controlling enzyme activity.

### 3.1 Metabolite‐Mediated Immune Signaling

Metabolites generated through dysregulated metabolic pathways function not merely as intermediates of energy metabolism but also as bioactive signaling molecules capable of modulating immune cell behavior. Within the TME, aberrant accumulation or depletion of key metabolites reshapes immune signaling networks, influencing immune activation, differentiation, and tolerance.

Excessive glycolytic flux in tumor cells, often driven by suppression of gluconeogenic enzymes, leads to glucose deprivation and lactate accumulation, thereby impairing glycolysis‐dependent effector functions of cytotoxic T cells and NK cells. Elevated lactate concentrations further suppress immune cell activity by inhibiting cytokine production and promoting immune exhaustion, ultimately facilitating immune escape [9, 10]. In parallel, altered amino acid availability and metabolic stress signals contribute to sustained activation of immunosuppressive pathways, such as STAT3 signaling, reinforcing immune checkpoint expression and immune tolerance [12, 13, 14, 15, 16, 17].

Together, these findings establish metabolites as critical mediators of immune signaling, positioning metabolic reprogramming as a direct driver of immune suppression rather than a secondary consequence of tumor growth. FBP1 deficiency, by enhancing aerobic glycolysis and lactate production, exemplifies how metabolic enzyme dysregulation drives metabolite‐mediated immune suppression, linking loss of gluconeogenic control to impaired antitumor immunity.

### 3.2 Metabolic Enzymes as Immune Regulators

Beyond their classical catalytic functions, metabolic enzymes increasingly emerge as noncanonical regulators of immune signaling and transcriptional control. Enzymes involved in glucose metabolism, including FBP1, act as molecular nodes that integrate metabolic status with immune regulatory pathways.

Loss or downregulation of FBP1 enhances aerobic glycolysis and promotes immune evasion by intensifying nutrient competition and reinforcing immune checkpoint signaling [5]. Mechanistically, FBP1 has been shown to regulate immune responses through enzymatic and nonenzymatic mechanisms, including modulation of transcription factor activity and immune checkpoint expression [5, 19]. In immune cells, alterations in metabolic enzyme expression influence polarization states and functional capacity, shaping innate and adaptive immune responses within the TME [9, 10].

These observations highlight metabolic enzymes as active participants in immune regulation, bridging intracellular metabolic states with immune signaling networks and functional outcomes. FBP1 serves as a paradigmatic example of such dual‐function metabolic enzymes, integrating its gluconeogenic catalytic activity with noncanonical regulatory functions that directly modulate STAT3 signaling, PD‐L1 expression, and macrophage polarization, thereby linking metabolic reprogramming to immune outcomes.

### 3.3 Intrinsic Regulatory Mechanisms of Metabolic Enzymes

The immune‐regulatory functions of metabolic enzymes are tightly controlled by intrinsic regulatory mechanisms that govern their expression, stability, and activity. Epigenetic modifications and posttranslational modifications enable tumor and immune cells to dynamically adjust enzyme function in response to environmental and inflammatory cues, thereby fine‐tuning immune outcomes.

#### 3.3.1 Epigenetic Regulation

Epigenetic modifications represent a major mechanism controlling metabolic enzyme expression in the TME. Promoter DNA methylation is a classical epigenetic regulatory process that frequently leads to transcriptional silencing. Aberrant methylation of the FBP1 promoter has been widely observed in multiple tumor types and is strongly associated with reduced gene expression and poor prognosis [20].

In NSCLC, hypermethylation of the FBP1 promoter significantly promotes tumor invasion and progression [21, 22]. In lung adenocarcinoma, upregulation of glycogen branching enzyme 1 (GBE1) induces abnormal FBP1 promoter methylation, facilitating malignant transformation [23]. Similarly, in cholangiocarcinoma, the long noncoding RNA DANCR recruits EZH2 to the FBP1 promoter, accelerating tumor progression through methylation‐dependent silencing [24]. In basal‐like breast cancer, transcription factor Snail enhances FBP1 promoter methylation, further driving disease aggressiveness [25]. Comparable epigenetic regulation has also been reported in hepatocellular carcinoma (HCC) and colorectal cancer [26, 27].

Importantly, pharmacological targeting of promoter methylation has demonstrated therapeutic potential. The small‐molecule compound CM‐272 effectively reduces FBP1 promoter methylation, restores its expression, and suppresses tumor progression in HCC models [28]. These findings underscore epigenetic regulation as a key determinant of metabolic enzyme–mediated immune modulation. FBP1 represents a well‐characterized example of how promoter hypermethylation silences metabolic enzyme expression in multiple cancer types, and the ability to therapeutically reverse this silencing highlights FBP1 as a tractable node for epigenetic intervention.

#### 3.3.2 Posttranslational Modifications

Posttranslational modifications (PTMs) provide an additional layer of control over metabolic enzyme function by regulating protein stability, localization, and molecular interactions. Phosphorylation and ubiquitination are particularly important PTMs influencing metabolic enzyme–immune crosstalk.

Phosphorylation of FBP1 exhibits tumor‐type–specific effects. In prostate cancer, PTEN loss leads to hyperphosphorylation of FBP1 via the PI3K/AKT pathway, accelerating protein degradation and promoting tumor progression [6]. In breast cancer, PIM2‐mediated phosphorylation at Ser144 enhances NF‐κB p65 activity, thereby driving proliferation, migration, and invasion [19]. Conversely, impaired phosphorylation can also compromise tumor‐suppressive functions. In HCC [7] and NSCLC [19], aberrant O‐GlcNAcylation of FBP1 disrupts PERK‐mediated phosphorylation at Ser170, abolishing its protective effects and facilitating tumor cell survival.

Ubiquitin‐mediated proteostasis further regulates metabolic enzyme abundance. In pancreatic cancer, E3 ubiquitin ligases TRIM47 [29] and UBR5 [30] promote ubiquitination and degradation of FBP1, whereas USP44 counteracts this process by stabilizing the protein [31]. In HCC, MAGE‐A3/C2 similarly enhances ubiquitin‐dependent degradation, while USP22 indirectly promotes tumorigenesis by modulating FBP1‐associated transcriptional complexes [32, 33]. Therapeutically, inhibition of aberrant ubiquitination using CDK4/6 inhibitor PD0332991 stabilizes FBP1 and suppresses tumor growth in PDAC [34].

**FIGURE 2:**
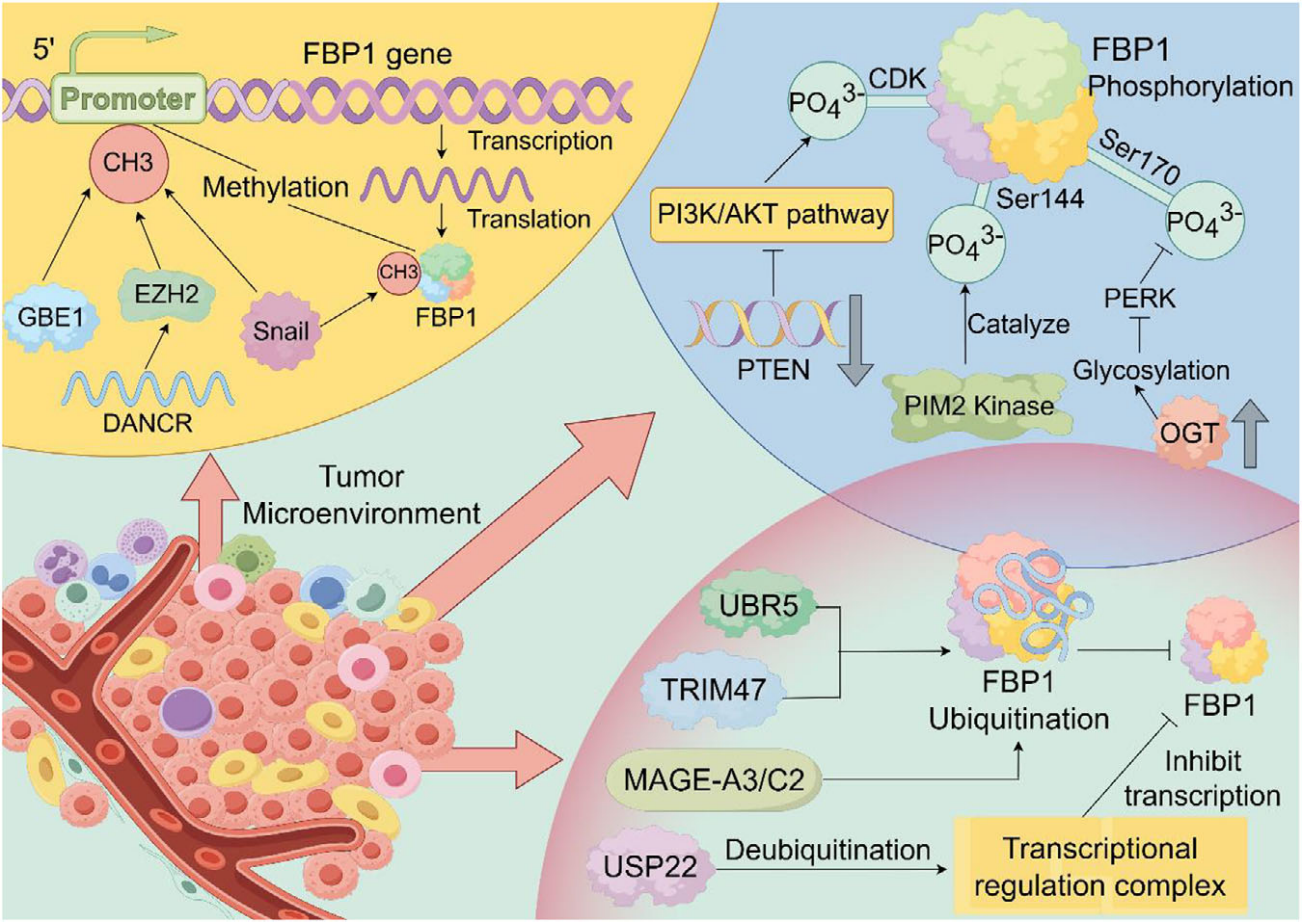
Regulatory mechanisms of FBP1 in the TME. From the transcriptional point of view, promoter methylation is an important factor in inhibiting the expression of the FBP1 gene. For example, GBE1 can directly induce promoter methylation, DANCR promotes methylation by recruiting EZH2, and Snail promotes not only promoter methylation but also FBP1 methylation. Phosphorylation modification of FBP1 also affects its activity and stability. For example, the downregulation of the tumor suppressor gene PTEN promotes phosphorylation of the CDK site of FBP1 by inhibiting the PI3K/AKT pathway, and PIM2 kinase can directly catalyze the phosphorylation of the Ser144 site, while the upregulation of OGT may hinder the phosphorylation of the Perk‐mediated Ser170 site through glycosylation modification. Ubiquitination modification is one of the important mechanisms leading to the decrease in FBP1 expression level. For example, UBR5, TRIM47, and MAGE‐A3/C2 reduced FBP1 levels by promoting ubiquitination modifications, while USP22, as a deubiquitinating enzyme, indirectly inhibited the expression of FBP1 by deubiquitinating FBP1‐related transcriptional regulatory complexes. FBP1, fructose‐1,6‐bisphosphatase 1; CDK, cyclin‐dependent kinase; PI3K, phosphoinositide 3‐kinase; AKT, protein kinase B; PTEN, phosphatase and tensin homolog; PIM2, proviral integration site for Moloney murine leukemia virus 2; PERK, protein kinase R‐like endoplasmic reticulum kinase; OGT, O‐linked N‐acetylglucosamine transferase; GBE1, glycogen branching enzyme 1; EZH2, enhancer of zeste homolog 2; DANCR, differentiation antagonizing non‐protein coding RNA; UBR5, ubiquitin protein ligase E3 component n‐recognin 5; TRIM47, tripartite motif containing 47; MAGE‐A3, melanoma‐associated antigen family member A3; MAGE‐C2, melanoma‐associated antigen family member C2; USP22, ubiquitin‐specific peptidase 22.

Together, these intrinsic regulatory mechanisms ensure precise control of metabolic enzyme function, enabling dynamic modulation of immune responses in the TME. Thus, the dysregulation of FBP1 expression is orchestrated not solely by singular genetic alterations but by an integrated network of epigenetic, posttranslational, and proteostatic mechanisms within the TME (Figure 2). Thus, FBP1 serves as a prime model for understanding how multilayered intrinsic regulatory mechanisms—encompassing epigenetic silencing, phosphorylation‐dependent stability control, and ubiquitin‐mediated proteostasis—converge to dictate metabolic enzyme expression and function, ultimately shaping metabolic‐immune crosstalk in cancer.

## 4 Metabolic Regulations of Immune Responses in Diseases

Metabolic reprogramming is a hallmark of tumor initiation and progression. As a key rate‐limiting enzyme in gluconeogenesis, FBP1 has recently been identified as playing important roles in metabolic regulation and immune modulation across various diseases, especially malignancies. This section systematically reviews the expression characteristics, regulatory mechanisms, and functional heterogeneity of FBP1 in multiple tumor types. By integrating perspectives from metabolic regulation, gene expression control, and TME remodeling, this section aims to elucidate the complex role of FBP1 as either a tumor suppressor or a context‐dependent oncogenic factor, and to explore its potential as a therapeutic target for clinical application. Structurally, each subsection focuses on a distinct cancer type, sequentially addressing the mechanisms regulating FBP1 expression, its impact on tumor biological behavior, its involvement in therapeutic resistance, and potential strategies for targeted intervention. Overall, this framework provides a systematic perspective for understanding both the common and context‐specific functions of FBP1 across different cancers.

### 4.1 Hepatocellular Carcinoma

HCC is among the most common primary liver cancers worldwide, with high incidence and mortality rates. As a key tumor suppressor, FBP1 regulates the biological behavior of HCC cells through complex, multidimensional mechanisms, and its loss or downregulation is strongly associated with HCC malignancy.

First, FBP1 modulates malignant phenotypes in HCC cells via metabolic pathways. Multiple studies have confirmed that FBP1 effectively inhibits HCC development [35], notably by reducing glucose uptake and aerobic glycolysis in tumor cells [4, 36]. In contrast, HCC cells gain metabolic advantages by downregulating FBP1: HCC cells inhibit FBP1 by utilizing LOXL2 [37], MAGE‐TRIM28 complex [32], and heat shock factor 2 (HSF2) [36], thereby reinforcing the Warburg effect, tumor cell proliferation, and invasive capabilities [3].

Second, gene expression regulatory changes also contribute to FBP1 loss and HCC progression. FBP1 dysregulation can promote virus‐mediated HCC progression. For example, high expression of the epigenetic reader protein Sp110 is associated with FBP1 and poor prognosis in hepatitis virus‐induced HCC [38]. Overexpression of OGT [7] and LIM domain and actin‐binding 1‐like protein (LIX1L) [39] in HCC can suppress FBP1, enhancing cell proliferation, migration, and invasion. At the transcriptional and translational levels, upregulation of miR‐517a [40], elevated expression of the helicase lymphoid‐specific protein (HELLS) [41], and increased activity of histone deacetylases HDAC1 and HDAC2 [42] all lead to significant decreases in FBP1 expression and accelerate HCC malignancy.

Critically, loss of FBP1 also profoundly reshapes the HCC TME. Reduced FBP1 expression not only suppresses NK cell‐mediated antitumor immune responses [43] but also accelerates tumor cell cycle G2‐M progression [44] and EMT [45]. Simultaneously, FBP1 deficiency activates HSCs and induces the SASP, disrupting hepatic metabolic homeostasis and further promoting tumor progression [46].

Based on these regulatory mechanisms, restoring FBP1 expression is emerging as a potential anticancer strategy. Factors and compounds that upregulate FBP1, such as the transcription factor FOXP2 [47], the small molecule CM‐272 [28], and CCAAT/enhancer binding protein alpha (C/EBPα) and hepatocyte nuclear factor 4α (HNF4α) [48], significantly induce HCC apoptosis and inhibit tumor growth and metastasis by increasing FBP1 levels.

In summary, FBP1 serves as a key tumor suppressor in HCC, inhibiting tumor development through metabolic modulation, gene regulation, and immune microenvironmental interactions. Its loss or repression triggers a series of malignant changes. Elucidating the mechanisms of FBP1 in HCC may facilitate the development of novel, more precise therapeutic targets for clinical liver cancer management.

### 4.2 Non–Small Cell Lung Cancer

Recent studies have demonstrated that downregulation of FBP1 is an independent risk factor for poor prognosis in non–small cell lung cancer (NSCLC) patients [22, 49, 50, 51]. FBP1 is also an important hypoxia‐associated gene [52, 53], and its downregulation promotes increased glycolytic activity and accelerates NSCLC progression. Low FBP1 expression both signifies and exacerbates tumor progression.

Mechanistically, FBP1 expression in NSCLC cells is suppressed by multiple factors [54]. The zinc‐finger transcription factor ZEB1 binds directly to the FBP1 promoter to inhibit transcription [55], while hypoxia‐induced upregulation of GBE1 increases FBP1 promoter DNA methylation, thereby reducing FBP1 expression [23]. Promoter methylation‐mediated FBP1 silencing further promotes EMT, markedly enhancing NSCLC cell proliferation and invasion [54]. Additionally, miR‐21a directly targets the 3'UTR of FBP1, inhibiting translation and driving NSCLC malignancy [56]. In models of hexavalent chromium [Cr(VI)]‐induced malignant transformation, FBP1 loss is also a key event in NSCLC carcinogenesis [57]. Immunologically, this low‐FBP1 state directly accelerates MDSC accumulation, converting the highly glycolytic niche into a barrier against effective T cell infiltration. These mechanisms collectively result in FBP1 functional deficiency, accelerating NSCLC aggressiveness and drug resistance.

Conversely, increasing FBP1 expression demonstrates significant antitumor potential in NSCLC therapy [58]. Restoration of FBP1 expression promotes ubiquitin‐mediated degradation of the NICD1, suppressing stem cell‐like properties in NSCLC cells [59]. The natural anticancer compound andrographolide upregulates FBP1, activates mitochondrial apoptotic pathways, and inhibits NSCLC‐cell proliferation [60]. High FBP1 expression can also reverse gefitinib resistance in NSCLC [50, 61] and induces apoptosis by suppressing telomerase reverse transcriptase (TERT) activity [62]. These findings suggest FBP1 restoration or enhancement as a promising strategy to inhibit NSCLC malignancy and improve therapeutic sensitivity.

### 4.3 Breast Cancer

FBP1 expression varies markedly between different subtypes of breast cancer (BC), reflecting its complex and diverse functions in breast tumorigenesis and progression. In triple‐negative breast cancer (TNBC), FBP1 is usually lost, marking metabolic reprogramming and the highly invasive phenotype of TNBC [63, 64, 65, 66], while in the progression from fibroadenomatous epithelial lesions (FELs) to malignancy, FBP1 is significantly upregulated. Brain metastases of BC also show higher secondary FBP1 expression compared with primary lesions [67]. Differences in FBP1 expression between benign fibroadenoma and malignant invasive BC have also been reported, though the dynamics and mechanisms require further investigation [68, 69]. These observations highlight the complexity of FBP1 regulation in breast pathologies, with its role differing according to clinical and pathological context.

In general, FBP1 upregulation exerts antitumor effects. For example, FBP1 upregulated through UCP1‐mediated mechanisms [70] can inhibit Wnt/β‐catenin pathway activity [71], block HIF‐1α signaling [72], mediate mitophagy to induce tumor cell apoptosis [73], and restrain BC cell proliferation and metastasis. Conversely, several mechanisms suppress FBP1 in BC cells, facilitating malignancy. For instance, KMT5A suppresses FBP1 via TWIST1 methylation, contributing to chemoresistance [74]. Moreover, downregulation of lncRNA SOX9‐AS1 [75] and HMGB2 [76] also promotes BC by targeting FBP1. Regarding the immune microenvironment, the loss of FBP1 in TNBC is linked to a shift toward aerobic glycolysis, which acidifies the TME and impairs the function of cytotoxic T lymphocytes and NK cells, thereby promoting an immune‐excluded phenotype.

However, in special contexts, such as adipocyte‐derived exosomal circCRIM1 [77] and during hypoxia or radiotherapy [78], FBP1 upregulation may paradoxically promote BC progression, possibly as a compensatory or adaptive response by tumor cells, though the exact mechanisms require further study.

Thus, FBP1 displays “dual roles” in BC: most research confirms its function as a multifaceted tumor suppressor, but some evidence indicates that upregulation under specific conditions may relate to tumor progression. Future BC‐targeted therapies based on FBP1 must consider tumor subtype, clinical context, and microenvironmental factors for optimal efficacy.

### 4.4 Clear Cell Renal Cell Carcinoma

Clear cell renal cell carcinoma (ccRCC) is the most common renal cancer subtype, with FBP1 identified as a key prognostic marker linked to ccRCC metabolic status [79, 80, 81, 82, 83]. FBP1 downregulation is associated with metabolic abnormalities, hypoxic microenvironment formation, and increased invasiveness in ccRCC [84]. Compared with normal tissue, RCC shows upregulation of glycolysis, pentose phosphate, glutamine metabolism, and lipid synthesis genes. Preclinical studies indicate that mutations in VHL, FBP1, and PI3K‐AKT‐mTOR pathways activate HIF transcriptional activity, driving aerobic glycolysis [85]. In ccRCC, loss of FBP1 exacerbates the pseudohypoxic state, leading to the accumulation of HIFs. This not only drives metabolic reprogramming but also promotes the recruitment of immunosuppressive cells such as TAMs and regulatory T cells (Tregs), while inhibiting the activation of effector T cells, creating a profoundly immunosuppressive milieu.

Restoring FBP1 expression markedly suppresses RCC progression [1]. At the molecular level, enhancers in the miR‐24‐1 region [86], DNA demethylase TET2 [87], and phosphofructokinase‐2,6‐bisphosphatase 4 (PFKFB4) [88] can upregulate FBP1, thereby inhibiting the Warburg effect and restraining ccRCC proliferation and metastasis. TET2 promotes FBP1 expression via promoter demethylation, implicating epigenetic regulation, while PFKFB4 underscores the metabolic network's influence on FBP1 function. These findings validate FBP1's tumor suppressive role in ccRCC and support combined metabolic and epigenetic strategies to restore FBP1 for ccRCC treatment [79].

### 4.5 Pancreatic Ductal Adenocarcinoma

Pancreatic ductal adenocarcinoma (PDAC) is a highly invasive malignancy with late diagnosis, rapid progression, frequent drug resistance, and extremely poor prognosis [89]. Recent studies show that FBP1 is generally downregulated in PDAC, a change closely linked to immune suppression and therapeutic resistance [5].

Loss of FBP1 may promote PDAC malignancy through various mechanisms. FBP1 deficiency disrupts the normal expression pattern of the key transcription factor c‐Myc, markedly increasing resistance to BET inhibitors [89]. Conversely, restoring or increasing FBP1 expression may overcome drug resistance. Recent research shows that deubiquitinases USP22 [33] and USP44 [31] stabilize and upregulate FBP1, reversing resistance to chemotherapeutics such as gemcitabine. FBP1 also significantly regulates PDAC sensitivity to PARP inhibitors, with FBP1 expression enhancing the antitumor efficacy of PARP inhibitors, suggesting its role as a potential therapeutic sensitizer [90].

Given the impact of FBP1 downregulation on PDAC malignancy, studies have focused on elucidating the molecular mechanisms underlying FBP1 loss. E3 ubiquitin ligases and chromatin regulatory proteins such as UBR5 [30], TRIM47 [29], and chromatin‐binding protein CBX3 [91] play key roles. USP7 can bind and deubiquitinate FBP1, preventing its nuclear translocation. USP7 inhibitors may thus increase nuclear FBP1, enhance sensitivity to PARP inhibitors, and provide significant synergistic antitumor effects [90]. USP25 is also implicated in pathological HIF‐1‐driven metabolic reprogramming and is a potential PDAC target [92]. These molecules reduce FBP1 protein levels via ubiquitin‐proteasome or epigenetic mechanisms, disrupting FBP1 interactions with key oncogenic proteins. Specifically, FBP1 binds the bromodomain of BRD4 [93] and the WW domain of IQGAP1 [94], inhibiting ERK1/2 phosphorylation and PDAC cell growth [95, 96]. E3 ligases or regulatory proteins disrupt these networks, resulting in loss of tumor suppression and promoting a more aggressive phenotype. Notably, PDAC tissues show significant upregulation of NPM1, correlating with poor prognosis; NPM1 inhibits FBP1 to activate aerobic glycolysis. Restoration of FBP1 can partially reverse NPM1's oncogenic effects, further linking FBP1 loss to poor PDAC outcomes [97]. Notably, FBP1 loss in PDAC is intrinsically linked to the establishment of an immunosuppressive environment. The resultant metabolic shift in cancer cells increases lactate secretion and suppresses the cytotoxic function of infiltrating T cells, contributing to the characteristic immune‐excluded TME of PDAC.

Pharmacological interventions targeting FBP1 regulation have shown promise. The CDK4/6 inhibitor PD0332991 (Palbociclib) effectively inhibits FBP1 degradation, stabilizes protein levels, and suppresses PDAC growth and invasion [34]. CDK4/6 inhibition can eliminate the glycolytic effect in PDAC by stabilizing FBP1 [98], highlighting the degradation pathway as a therapeutic target [99].

In summary, a dual intervention strategy—directly stabilizing/increasing FBP1 with drugs such as PD0332991, and inhibiting upstream negative regulators (e.g., UBR5 or TRIM47) to restore FBP1 interactions—may effectively reverse PDAC malignancy and enhance clinical efficacy [100, 101]. Targeting FBP1 regulation represents a promising breakthrough for PDAC therapy.

### 4.6 Colorectal Cancer

Recent research has shown marked downregulation of FBP1 in colorectal cancer (CRC), closely associated with CRC initiation and progression. Multiple factors suppress FBP1 in CRC, including loss of the mixed lineage leukemia fusion gene AF9 [102], decreased circFNDC3B circular RNA [103], and repression of FBP1 transcription by Forkhead box transcription factor C1 (FOXC1) [104]. For example, miR‐145 targets the 3'UTR of AF9 to inhibit its expression; loss of AF9 reduces gluconeogenic gene expression—including phosphoenolpyruvate carboxykinase 2 (PCK2) and FBP1—promoting glucose consumption and tumorigenesis. Thus, AF9 is crucial for upregulating PCK2 and FBP1, and the miR‐145/AF9 axis may be a promising CRC therapeutic target.

FBP1 exerts tumor suppressive effects by modulating multiple signaling pathways. It dephosphorylates IκBα protein [105], reduces NF‐κB pathway activity, and thereby inhibits tumor cell proliferation, inflammation, and invasion. FBP1 also induces G2‐M phase cell cycle arrest [27], restraining rapid proliferation and malignancy. When FBP1 is downregulated, these suppressive pathways fail, leading to uncontrolled proliferation, increased survival, and CRC progression. In the context of the CRC immune microenvironment, FBP1 downregulation contributes to a pro‐inflammatory state through NF‐κB activation, which can paradoxically promote the recruitment of MDSCs and TAMs, fostering an immunosuppressive environment that dampens effective antitumor immunity.

In other words, FBP1 downregulation removes key “braking mechanisms,” granting CRC cells greater invasiveness and malignancy. Thus, FBP1 is not only a potential biomarker for tumorigenesis but also a promising therapeutic target.

Increasing FBP1 expression or activity in CRC may restore its tumor‐suppressive functions—reactivating IκBα dephosphorylation, restoring cell cycle arrest, and limiting CRC progression. Strategies targeting upstream regulators such as AF9, circFNDC3B, and FOXC1 may restore FBP1 function, offering new therapeutic approaches and drug targets for CRC. These findings provide a foundation for new therapies and further research on FBP1‐related pathways in cancer.

### 4.7 Glioblastoma Multiforme

Glioblastoma multiforme (GBM) is the most common and aggressive primary brain tumor, characterized by high heterogeneity, invasiveness, and therapeutic resistance. Recent studies indicate that FBP1, a key rate‐limiting enzyme in gluconeogenesis, is significantly downregulated in GBM, closely related to metabolic reprogramming and immunosuppressive TME, suggesting FBP1 as a central metabolic and immune hub in GBM progression.

Specifically, GBE1 is markedly upregulated in GBM, directly suppressing FBP1 expression through transcriptional or posttranslational mechanisms [106]. Downregulation of FBP1 causes a metabolic shift favoring aerobic glycolysis, increasing glucose uptake and lactate production to fuel rapid proliferation and invasion [107]. This metabolic reprogramming is a key driver of the profoundly immunosuppressive TME in GBM. The accumulation of lactate and other oncometabolites not only fuels rapid tumor growth but also suppresses the function of infiltrating T cells and microglia, polarizing them toward a tumor‐supportive phenotype and facilitating immune escape [9, 108].

Therapeutic strategies based on FBP1 modulation are highly attractive. Theoretically, increasing FBP1 expression exerts antitumor effects through two mechanisms: (1) blocking aberrant glycolysis and limiting glucose exploitation for rapid growth; (2) alleviating metabolic immunosuppression, restoring the activity and function of immune cells (e.g., infiltrating T cells, microglia), and enhancing antitumor immunity.

Therefore, FBP1‐targeted therapies—including pharmacological activation, gene editing, or inhibition of negative regulators such as GBE1—represent highly promising directions for GBM treatment. Further investigation of FBP1‐mediated metabolic‐immune regulation and development of FBP1‐centric combinatory therapies may significantly improve GBM outcomes and patient survival.

### 4.8 Prostate Cancer

The function of FBP1 in prostate cancer (PCa) has recently garnered increasing attention, though its mechanisms are complex and dualistic. Substantial evidence indicates that FBP1 exhibits both tumor‐suppressive and oncogenic properties during PCa progression, showing context‐dependent effects.

On one hand, FBP1 is a crucial downstream effector in the PTEN (Phosphatase and Tensin Homolog Deleted on Chromosome 10) pathway. In normal prostate tissue or early‐stage tumors, PTEN maintains high FBP1 expression to suppress tumorigenesis. PTEN mutation or loss reduces FBP1 levels, undermining its tumor‐suppressive capacity and promoting PCa cell proliferation, invasion, and metastasis [6]. These data highlight the necessity of maintaining FBP1 expression to prevent PCa progression. In the early stages, this FBP1‐mediated metabolic control likely contributes to the immune environment that inhibits tumor growth.

On the other hand, FBP1 also influences PCa treatment sensitivity. Notably, FBP1 depletion triggers tumor‐suppressive AMPK‐mTOR‐autophagy axis activation and promotes DNA damage‐mediated apoptosis, thereby enhancing PCa sensitivity to radiotherapy [109], while TRIM47‐mediated FBP1 induction drives protumorigenic metabolic rewiring via Warburg effect potentiation in PCa [110]. These pivotal findings illuminate that high FBP1 expression may thus confer survival advantages and treatment refractory to PCa, thus posing new challenges for radiotherapy. This context‐dependent duality extends to immune modulation; in advanced or treatment‐resistant PCa, high FBP1 expression may be associated with a metabolically hostile microenvironment that excludes immune cell infiltration, contributing to the “cold” tumor phenotype typically observed in this disease.

Overall, FBP1 in PCa is context‐dependent—acting as a tumor suppressor by maintaining normal metabolism under some conditions, but potentially promoting malignancy and resistance in others. This duality of functions has a central impact on the formulation of its treatment strategy. At the clinical level, this means that FBP1 cannot simply be considered as a universal therapeutic target. The clinical interpretation of its expression level must be closely related to the molecular subtype, stage, and treatment history of the disease. For example, in treatment‐naïve localized PCa, strategies aimed at restoring or activating FBP1 function may have the benefit of preventing tumor progression. Conversely, FBP1 function needs to be carefully evaluated in advanced patients who have developed treatment resistance, where targeting its downstream effector pathways or combination strategies with specific chemotherapy/targeted agents may be more effective. Therefore, future research focuses on elucidating key signals that drive FBP1 functional transition and developing biomarkers based on FBP1 functional status to guide personalized treatment stratification.

### 4.9 Gastric Cancer

FBP1 expression in gastric cancer (GC) is primarily regulated at the epigenetic level, most commonly through promoter hypermethylation. Studies have shown that FBP1 is significantly downregulated in GC compared with normal gastric mucosa, correlating strongly with promoter hypermethylation [111]. As a key enzyme in gluconeogenesis, FBP1 downregulation promotes glycolytic metabolism in GC cells. This metabolic remodeling provides energy for EMT, thereby enhancing migration and invasion [112]. Thus, suppression of FBP1 is an essential adaptation for more aggressive GC cell phenotypes.

However, in the complex GC microenvironment, FBP1 may not always act as a tumor suppressor. In certain situations, it may even exhibit protumor potential, particularly in immune regulation. Recent studies have shown that GC‐associated mesenchymal stem cells (GC‐MSCs) can upregulate FBP1 to suppress NK cell‐mediated antitumor immunity [113]. Specifically, MSCs enhance FBP1 to inhibit NK cell metabolism, reduce cytotoxicity, and allow immune evasion. This highlights how components of the GC microenvironment can “hijack” FBP1 to promote immune escape. This represents a unique paradigm where FBP1 upregulation directly suppresses the antitumor immune response, contrasting with its tumor‐suppressive role when expressed in cancer cells.

Understanding FBP1's diverse roles in tumor and microenvironmental cells is crucial for precision GC therapy. Therapeutic strategies must consider this functional complexity by reactivating FBP1 in tumor cells to restore tumor suppression, while blocking its immunosuppressive effects in the microenvironment. A comprehensive, dual‐targeting approach may maximize FBP1's therapeutic benefit and effectively curb GC progression.

### 4.10 Cholangiocarcinoma

FBP1 is significantly downregulated in cholangiocarcinoma (CCA) and is recognized as a key molecular marker of CCA initiation and progression. Multiple molecular mechanisms and signaling pathways are involved in FBP1 suppression. For example, reduced expression of long noncoding RNA MT1JP impairs FBP1 transcription [114], while lncRNA DANCR recruits histone methyltransferase EZH2 to the FBP1 promoter, inducing promoter methylation and transcriptional silencing [24]. Additionally, the E3 ubiquitin ligase NEDD4 accelerates FBP1 protein degradation via the ubiquitin‐proteasome pathway, reducing stability and expression in CCA cells [115].

FBP1 downregulation profoundly impacts CCA biology. Suppression of FBP1 weakens the antitumor Wnt/β‐catenin pathway, removing constraints on proliferation, invasion, and migration, and facilitating malignant progression [116]. From an immunological standpoint, the loss of FBP1 in CCA is associated with epigenetic reprogramming that not only promotes tumor cell proliferation but also contributes to a desmoplastic and immunosuppressive stroma, limiting the infiltration and function of effector T cells. Thus, restoring FBP1 expression holds great potential as a therapeutic strategy for CCA.

Notably, pharmacological modulation of FBP1 expression is showing promise. For example, the EZH2 inhibitor Tazemetostat can reverse promoter methylation, significantly increase FBP1 mRNA and protein levels, and exert marked antitumor effects [117]. This not only clarifies FBP1 regulation but offers new hope for CCA therapy—epigenetically or posttranslationally restoring FBP1's anticancer function. This epigenetic intervention, by restoring FBP1, holds the potential to simultaneously inhibit tumor cell proliferation and remodel the immune microenvironment toward a more antitumor state.

In sum, targeted interventions to reverse FBP1 downregulation—such as using EZH2 inhibitors like Tazemetostat, modulating lncRNAs MT1JP and DANCR, or inhibiting E3 ligase NEDD4—may be effective therapeutic strategies for CCA. As research into the FBP1 regulatory network progresses, more precise, efficient CCA‐targeted agents based on the FBP1 pathway may be developed, enabling individualized therapy and improved outcomes for CCA patients.

### 4.11 Ovarian Cancer

Currently, there is no consensus regarding the role of FBP1 in the initiation, progression, and chemoresistance of ovarian cancer (OC), with significant discrepancies and controversies among different studies.

On one hand, some studies have demonstrated that FBP1 may exert oncogenic effects in OC. For example, it has been reported that high expression of FBP1 accelerates the cell cycle progression of OC cells, significantly promotes cellular migration, invasion, and distant metastasis, thereby facilitating the malignant progression of OC [118]. These findings suggest that, under certain conditions, FBP1 may endow cancer cells with greater invasiveness and survival advantages. Furthermore, studies employing FBP1 knockdown have shown that suppression of FBP1 significantly inhibits the EZH2/H3K27me3 signaling pathway, thereby reducing malignancy and attenuating cisplatin resistance in OC cells [119]. These findings further substantiate the potential positive role of FBP1 in promoting tumor survival and enhancing chemoresistance.

On the other hand, opposing evidence also exists. Recent research has revealed that when FBP1 expression is inhibited due to promoter methylation and C‐MYC binding, downregulated FBP1 promotes OC proliferation, metastasis, and cisplatin resistance by regulating STAT3 [120]. These results suggest that, under therapeutic pressure or specific contexts, FBP1 may possess tumor‐suppressive and chemosensitizing activities. This functional controversy extends to the immune microenvironment. It is plausible that in the chemoresistant setting, the metabolic status dictated by FBP1 expression could differentially impact the infiltration and activity of immune cells, such as TAMs and T cells.

Therefore, the current conclusions regarding the specific role of FBP1 in OC are contradictory, and its functions in tumor progression and chemoresistance require further clarification. A plausible explanation is that FBP1 may play a context‐dependent dual regulatory role in OC: its biological effects are regulated by multidimensional factors such as OC molecular subtypes, tumor progression stage, and treatment exposure background. Specifically, in untreated OC models, FBP1 may promote tumor cell proliferation and invasion by remodeling cellular metabolic pathways, such as enhancing glycolytic flux or regulating bioanabolism. In the stress microenvironment induced by chemotherapy drugs such as cisplatin, the upregulation of FBP1 expression may activate the apoptosis signaling pathway or inhibit resistance‐related mechanisms, thereby enhancing chemotherapy sensitivity and promoting tumor cell death. This functional plasticity suggests that FBP1 is not a single oncogene or tumor suppressor gene in OC, but acts as a metabolic hub to achieve dynamic functional transformation in different physiological and therapeutic stress states of tumors.

### 4.12 Acute Myeloid Leukemia

The critical role of FBP1 in hematologic malignancies has garnered increasing attention, with FBP1 considered to possess distinct therapeutic potential. In studies on acute myeloid leukemia (AML), FBP1 expression levels have been strongly associated with disease progression and therapeutic sensitivity, positioning it as a potential therapeutic target [121]. For instance, it has been shown that active vitamin D3 (1,25‐dihydroxyvitamin D3, VD3) can markedly induce FBP1 upregulation in AML cells [122]. Additionally, VD3 can significantly improve mitochondrial metabolic function, reprogram energy utilization patterns, and activate mitochondria‐mediated apoptotic pathways, thereby fundamentally impairing the survival advantage of leukemic cells [123].

Moreover, Japanese research teams have reported that the oncogene Evi1 can directly regulate FBP1 expression; Evi1 upregulates FBP1 and activates the pentose phosphate pathway (PPP), leading to metabolic remodeling and driving AML progression [124]. This “Evi1‐FBP1‐PPP” axis represents a novel regulatory mechanism and a promising therapeutic target. This important finding not only highlights the pivotal role of FBP1 in AML metabolic regulation but also introduces the complete “Evi1‐FBP1‐PPP” metabolic axis as a focus for future research. Whether FBP1‐mediated metabolic reprogramming of AML cells in the bone marrow microenvironment modulates the activity of immune cells such as T cells and macrophages, thereby altering the TME, remains to be further explored.

Overall, AML is highly dependent on metabolic abnormalities. FBP1 expression levels are closely related to AML cell proliferation and differentiation. Elevated FBP1 expression is typically associated with slowed or arrested proliferation and induction of cellular differentiation and maturation, indicating that FBP1 can serve as a positive regulator in AML suppression. Based on current research, pharmacological interventions such as VD analogs have demonstrated potent antileukemic effects both in vitro and in vivo by upregulating FBP1 [125]. This suggests that future clinical strategies targeting FBP1, including differentiation‐inducing therapies, may have significant translational value.

### 4.13 Esophageal Cancer

FBP1 has been identified as a key molecule with important prognostic and functional significance in esophageal cancer (EC), exhibiting distinct expression patterns and functions across different subtypes.

In esophageal adenocarcinoma (EAC), high FBP1 expression is significantly correlated with favorable patient prognosis and serves as an independent and advantageous prognostic marker. Clinical observations indicate that EAC patients with high FBP1 expression generally experience slower tumor progression, reduced metastatic potential, and longer disease‐free and overall survival [126, 127]. In contrast, in esophageal squamous cell carcinoma (ESCC), FBP1 expression is markedly decreased or absent [128]. The loss of FBP1 significantly enhances fatty acid metabolism, promotes proliferation, migration, and invasion of ESCC cells, and accelerates tumor progression. Recent studies have further revealed that this loss is closely associated with microRNA regulation, particularly with high expression of miR‐18b‐5p, which strongly inhibits FBP1 levels. Notably, miR‐18b‐5p inhibitors can effectively restore FBP1 expression and reverse malignant phenotypes, thereby suppressing ESCC progression [129]. Although direct evidence is limited, the distinct metabolic profiles resulting from FBP1 loss in ESCC versus its retention in EAC likely contribute to divergent immune microenvironments, potentially explaining the differential aggressiveness and therapeutic responses between these two subtypes.

These findings indicate that FBP1 exerts significant tumor‐suppressive effects in both esophageal adenocarcinoma and ESCC. High FBP1 expression correlates with reduced tumor aggressiveness, higher differentiation, and improved patient prognosis, whereas low or absent FBP1 expression suggests increased malignancy and poor prognosis. Therefore, FBP1 may serve as an important prognostic biomarker and potential therapeutic target in EC [130]. Future studies may explore upregulation of FBP1 via microRNA modulation, combined with fatty acid metabolism inhibitors and other metabolic interventions, to synergistically enhance FBP1's antitumor activity and provide new therapeutic avenues for EC.

### 4.14 Other Cancers

Beyond the aforementioned types, the role of FBP1 in numerous other cancers has been reported. Generally, low FBP1 expression is associated with poor prognosis. For example, in bladder cancer, decreased expression of FBP1 is not only associated with poor clinical prognosis, but also promotes the proliferation and metastasis of cancer cells, and the mechanism may be related to glucose metabolism reprogramming [11, 131]. In osteosarcoma, studies have shown that the absence of FBP1 expression significantly enhances tumor aggressiveness and leads to reduced survival, suggesting its value as a potential prognostic marker [132, 133]. In addition, in cervical cancer, the low expression status of FBP1 is associated with HPV infection and tumor stage progression, further highlighting its regulatory role in cancer development [134]. Studies of head and neck squamous cell carcinoma [135] and papillary thyroid carcinoma [136] have also shown that downregulation of FBP1 expression not only predicts poor prognosis but also participates in key metabolism processes such as glycolysis, apoptosis, and HIF‐1α. A common thread across these diverse cancers is that FBP1 downregulation drives a glycolytic shift, which is a well‐established mechanism for creating an immunosuppressive TME, characterized by impaired effector T cell function and increased recruitment of immunosuppressive cell populations. On the contrary, increasing FBP1 expression helps inhibit tumor progression. For example, in nasopharyngeal carcinoma, increasing FBP1 expression can effectively inhibit tumor growth and enhance radiosensitivity, suggesting that it may be a new target for the treatment of this disease [137]. These studies jointly show that FBP1 has a wide range of tumor‐suppressive functions in a variety of cancers, and its expression level is closely related to tumor progression and patient prognosis, providing an important basis for the development of targeted intervention strategies in the future.

**FIGURE 3:**
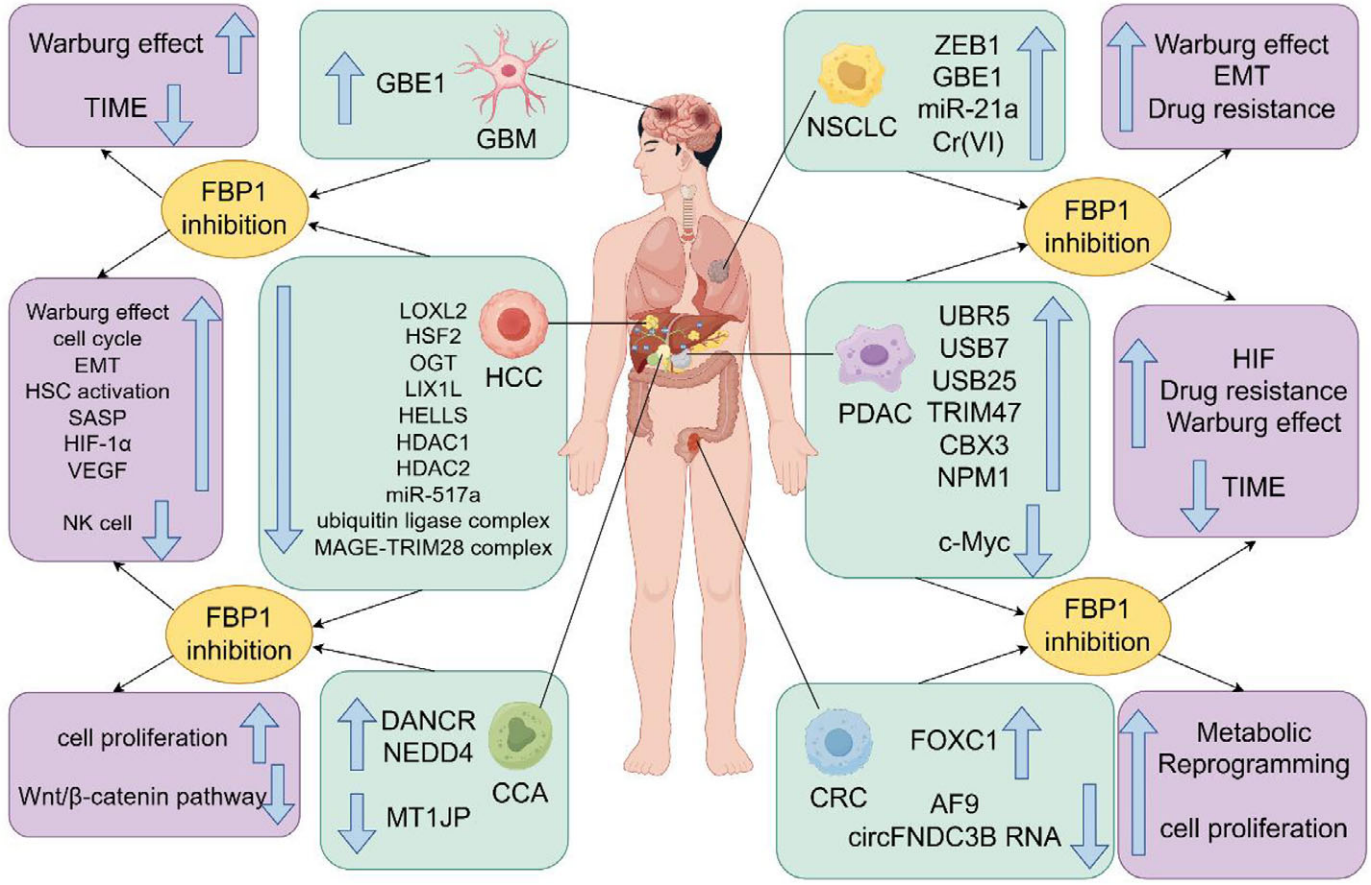
Specific mechanisms and oncogenic effects of FBP1 downregulation in tumor cells. In GBM, elevated GBE1 suppresses FBP1 expression, thereby promoting aerobic glycolysis and inhibiting the TME. In HCC, the downregulation of factors such as LOXL2, HSF2, OGT, LIX1L, HELLS, HDAC1, HDAC2, miR‐517a, ubiquitin ligase complex, and MAGE‐TRIM28 complex further suppresses FBP1, subsequently facilitating aerobic glycolysis, cell cycle progression, EMT, HSC activation, and the downregulation of regulatory factors, including SASP, HIF‐1α, and VEGF, while also inhibiting the NK cell‐mediated immune microenvironment. In CCA, increased expression of DANCR and NEDD4, as well as decreased MT1JP, inhibits FBP1 function, thereby accelerating tumor cell proliferation and impeding the tumor‐suppressive effect of the Wnt/β‐catenin pathway. In NSCLC, upregulation of ZEB1, GBE1, miR‐2a, and Cr(VI) inhibits FBP1 through distinct mechanisms, promoting aerobic glycolysis, EMT, and tumor chemoresistance. In PDAC, upregulation of regulatory factors UBR5, USP7, USP25, TRIM47, CBX3, and NPM1, as well as downregulation of c‐Myc, all suppress FBP1 expression, thus facilitating aerobic glycolysis, chemoresistance, enhanced HIF activity, and inhibition of the TME. In CRC, upregulation of FOXC1 or suppression of AF9 and circFNDC3B RNA can inhibit FBP1, thereby promoting metabolic reprogramming and cell proliferation. AF9, ALL1‐fused gene from chromosome 9; CBX3, chromobox 3; CCA, cholangiocarcinoma; circFNDC3B, circular RNA fibronectin type III domain containing 3B; Cr(VI), chromium(VI); c‐Myc, cellular myelocytomatosis oncogene; DANCR, differentiation antagonizing non‐protein coding RNA; EMT, epithelial‐mesenchymal transition; FOXC1, forkhead box C1; GBE1, glycogen branching enzyme 1; GBM, glioblastoma multiforme; HCC, hepatocellular carcinoma; HDAC1, histone deacetylase 1; HDAC2, histone deacetylase 2; HELLS, helicase, lymphoid‐specific; HIF, hypoxia‐inducible factor; HIF‐1α, hypoxia‐inducible factor‐1α; HSC, hepatic stellate cell; HSF2, heat shock transcription factor 2; LOXL2, lysyl oxidase‐like 2; LIX1L, LIX1 like; MAGE, melanoma‐associated antigen; miR‐21a, microRNA‐21a; miR‐517a, microRNA‐517a; MT1JP, metallothionein 1J pseudogene; NEDD4, neural precursor cell expressed developmentally down‐regulated 4; NK cell, natural killer cell; NPM1, nucleophosmin 1; NSCLC, non–small cell lung cancer; OGT, O‐linked N‐acetylglucosamine transferase; PDAC, pancreatic ductal adenocarcinoma; SASP, senescence‐associated secretory phenotype; TRIM28, tripartite motif containing 28; TRIM47, tripartite motif containing 47; UBR5, ubiquitin protein ligase E3 component n‐recognin 5; VEGF, vascular endothelial growth factor; ZEB1, zinc finger E‐box binding homeobox 1.

It is noteworthy that, although FBP1 predominantly acts as a tumor suppressor, its function may vary or even reverse in certain tumors or specific contexts. Therefore, before universally targeting FBP1 in therapy, it is essential to delineate the molecular background and FBP1‐related pathways in different tumor types. Therapeutic strategies targeting FBP1‐mediated immunometabolic pathways hold potential to reverse immune suppression and enhance treatment response across diverse cancer types (Table 2). Consequently, FBP1 downregulation is now recognized not merely as a metabolic defect but as a tumor‐type‐specific driver of malignancy, orchestrating immune evasion and therapeutic resistance through diverse molecular mechanisms (Figure 3).

**TABLE 2:** Molecular regulatory mechanisms of FBP1 in various tumor cells and their prognostic and therapeutic value.

Tumor cell	FBP1 expression	Molecular regulatory mechanisms	Prognostic evaluation	Therapeutic implications
HCC	Decrease	Regulate aerobic glycolysis [2, 43, 99] and gene expression levels [6, 23, 62, 138, 139, 140]	A prognostic biomarker in predictive models [2]	Support therapeutic strategies aimed at restoring FBP1 expression [47, 141]
NSCLC	Decrease	Promoter methylation [10, 142] suppresses cancer‐cell apoptosis [35, 59, 123]	An independent prognostic marker [9, 143, 144, 145, 146] in multigene prognostic models [10, 63]	
BC	Subtype‐dependent	Regulate signaling pathways [48], mitochondrial autophagy [35], and metabolic reprogramming [7, 19]	Provide potential diagnostic and prognostic value	Targeted regulation of FBP1 offers additional strategies [22, 42, 51, 147]
ccRCC	Decrease	Regulate hypoxia‐associated genes [58] and metabolic reprogramming [50, 60, 61]	A prognostic biomarker for predicting long‐term survival [3, 53, 54, 55, 56]	
PDAC	Decrease	Regulate immune cell infiltration, immune response [5], and tumor drug resistance [64, 119]	Predict patient prognosis [22, 54, 65]	Provides new strategies to overcome chemoresistance
CRC	Decrease	Regulate cell cycle checkpoints [76] and influence tumor cell proliferative rate	An independent prognostic marker in predictive models [66, 67, 68]	
GBM	Decrease	Regulate immune response, aerobic glycolysis, TME [71, 74], and metabolic reprogramming [70]	A prognostic biomarker [71, 74] in multigene prognostic models	
PCa	Decrease	Regulate gene expression [75], apoptosis [93], and DNA damage repair mechanisms [93]	A potential biomarker for predicting radiotherapy response	Targeted regulation modulates radiosensitivity [93] and response
GC	Decrease	Regulate glycolysis [25] and antitumor immune responses [77]	A prognostic biomarker to predict survival [11, 76]	Targeted regulation enhances immunotherapy efficacy [77]
CCA	Decrease	Epigenetic regulation and promoter methylation [44]	Predict prognosis of oncology patients [80]	Aids in targeted therapies.
AML	Decrease	Regulate aerobic glycolysis, energy metabolism [84], and apoptosis [110]	Applied in glycolysis‐based prognostic models [83]	Potential therapeutic target in antitumor therapy
EC	Decrease	Regulate fatty acid metabolism and cell proliferation [37]	An independent biomarker [85] in prognostic models	Provide new perspectives for targeted therapy

Abbreviations: AML, acute myeloid leukemia; BC, breast cancer; CCA, cholangiocarcinoma; ccRCC, clear cell renal cell carcinoma; CRC, colorectal cancer; EC, esophageal cancer; GBM, glioblastoma multiforme; GC, gastric cancer; HCC, hepatocellular carcinoma; NSCLC, non–small cell lung cancer; PCa, prostate cancer; PDAC, pancreatic ductal adenocarcinoma.

## 5 Immunometabolic Mechanisms and Therapeutic Targeting in Cancer

Cancer‐associated metabolic reprogramming actively reshapes the tumor immune microenvironment, influencing immune cell function, immune checkpoint signaling, and therapeutic responsiveness. Dysregulated metabolic pathways and enzymes modulate nutrient competition, metabolite signaling, and immune cell differentiation, thereby promoting immune evasion and resistance to immunotherapy. These alterations represent actionable immunometabolic vulnerabilities rather than passive byproducts of tumor growth. Targeting key metabolic nodes provides a mechanistically grounded strategy to restore antitumor immunity and enhance the efficacy of immune‐based therapies across diverse cancer contexts. Among these nodes, FBP1 has emerged as a central immunometabolic regulator, orchestrating cross‐talk between tumor cells, immune cells, and stromal components through both its canonical metabolic activity and noncanonical protein–protein interactions.

### 5.1 Targeting the FBP1‐STAT3 Axis to Overcome PD‐L1‐Driven Immune Evasion and Therapy Resistance

Immune checkpoint signaling represents a central mechanism by which tumors evade immune surveillance, with the PD‐1/PD‐L1 axis playing a dominant role in suppressing T‐cell activation and cytotoxic function [14, 15, 16, 17]. Accumulating evidence indicates that immune checkpoint expression is tightly regulated by intracellular metabolic states and metabolic enzymes, linking tumor metabolic reprogramming to immune escape. Among key regulators, STAT3 serves as a metabolic‐responsive transcription factor whose persistent activation promotes PD‐L1 transcription and tumor progression [12, 13, 148, 149, 150, 151, 152, 153, 154, 155].

Recent studies have identified metabolic enzymes as noncanonical regulators of immune checkpoint signaling. Specifically, FBP1 functions as an endogenous suppressor of PD‐L1 expression by competitively binding unphosphorylated STAT3 and limiting its transcriptional access to the PD‐L1 promoter, independent of its enzymatic activity [5]. This metabolic–transcriptional coupling has been observed across multiple cancer types, including pancreatic and prostate cancers, supporting a conserved immunometabolic mechanism through which metabolic dysregulation directly licenses immune checkpoint activation.

**FIGURE 4:**
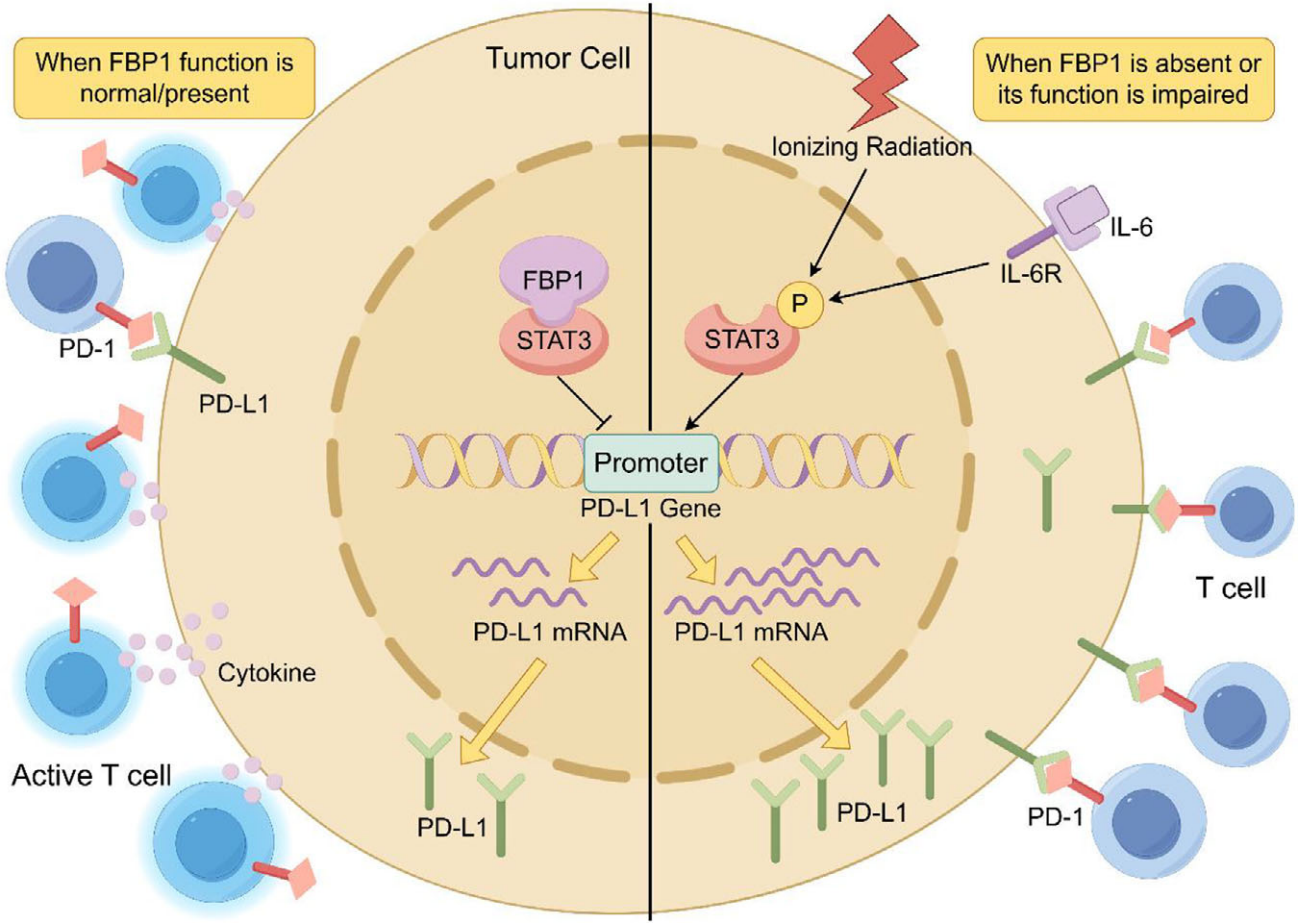
FBP1 negatively regulates PD‐L1 expression by antagonizing STAT3 transcriptional activity. When FBP1 is functional/present, within the tumor nucleus, FBP1 binds to unphosphorylated STAT3 to form a complex. This complex prevents STAT3 from binding to the promoter region of the PD‐L1 gene, resulting in low levels of mRNA and protein expression of PD‐L1. When cytotoxic T cells approach, the PD‐1 receptors on the surface cannot find enough PD‐L1 ligands to bind, causing T cells to remain activated, release cytokines, and kill tumor cells. Ionizing radiation or IL‐6 binds to the on‐membrane receptor IL‐6R, triggering intracellular STAT3 phosphorylation, and when FBP1 is absent or impaired, the phosphorylated STAT3 binds to the promoter, resulting in a significant increase in PD‐L1 mRNA and protein expression levels. PD‐1 on the surface of T cells successfully binds to PD‐L1 with high expression, causing T cells to be depleted and antitumor function to be inhibited. IL‐6, interleukin‐6; IL‐6R, interleukin‐6 receptor; mRNA, messenger RNA; PD‐1, programmed death‐1; PD‐L1, programmed death‐ligand 1; STAT3, signal transducer and activator of transcription 3.

Importantly, metabolic and inflammatory cues dynamically modulate this regulatory axis. Ionizing radiation or IL‐6 stimulation promotes STAT3 Y705 phosphorylation, disrupting the FBP1–STAT3 interaction and restoring STAT3‐dependent PD‐L1 transcription [5]. Clinically, inverse correlations between FBP1 and PD‐L1 expression have been documented in patient tumor specimens, and loss of FBP1 confers resistance to PD‐1/PD‐L1 blockade in preclinical models. Thus, these findings establish metabolic regulation of immune checkpoint signaling as a critical determinant of immune evasion and therapeutic response [18]. Therefore, FBP1 acts as a transcriptional decoy for unphosphorylated STAT3, thereby suppressing PD‐L1 promoter activity and enhancing T cell‐mediated cytotoxicity, whereas FBP1 loss or STAT3 phosphorylation licenses PD‐L1‐driven immune evasion (Figure 4).

Beyond transcriptional control, metabolic enzymes can also regulate immune checkpoint signaling through posttranslational and epigenetic mechanisms. For example, oncogenic kinases such as PIM2 phosphorylate metabolic regulators, impairing their inhibitory feedback on NF‐κB signaling and thereby indirectly enhancing PD‐L1 expression [19]. Similar regulatory patterns have been reported across lung cancer, pancreatic cancer, and glioma, suggesting that metabolic‐immune checkpoint coupling represents a broadly conserved mechanism rather than a tumor‐type‐restricted phenomenon [9, 156, 157].

### 5.2 Metabolic Modulation of Innate Immune Surveillance

Innate immune surveillance constitutes the first line of defense against tumor initiation and progression, and its efficacy is highly dependent on metabolic fitness. Emerging evidence indicates that tumor‐associated metabolic reprogramming profoundly reshapes the function of innate immune cells, particularly NK cells and macrophages, thereby facilitating immune escape. Alterations in metabolic enzymes and metabolite availability within the TME can impair innate immune cytotoxicity and promote immunosuppressive phenotypes. As a key regulator of gluconeogenesis and a tumor suppressor frequently lost in cancers, FBP1 plays a critical role in this process, mediating non‐cell‐autonomous metabolic crosstalk that suppresses innate immunity.

#### 5.2.1 NK Cell‐Mediated Antitumor Activity

NK cell–mediated antitumor activity is tightly coupled to glycolytic metabolism. In a KRAS‐driven lung cancer model, dynamic changes in tumor cell metabolism during progression were shown to suppress NK cell function, despite effective immune surveillance at early stages [158]. FBP1 loss in tumor cells is a central driver of this immunosuppressive metabolic communication. In HCC, loss of FBP1 in tumor cells alters extracellular vesicle (EV) composition, leading to reduced levels of pyruvate kinase L/R (PKLR). Uptake of these EVs by NK cells compromises their metabolic capacity and cytotoxic function, thereby promoting tumor initiation and progression [43]. These findings indicate that tumor metabolic rewiring can indirectly disable NK cell surveillance through metabolic crosstalk, highlighting a non‐cell‐autonomous mechanism by which tumor metabolism suppresses innate immune surveillance.

In addition to direct metabolic crosstalk, tumor angiogenic status and hypoxia further modulate NK cell activity by shaping nutrient and oxygen availability. Endothelial glycolysis‐dependent angiogenesis has been shown to reinforce hypoxic niches that impair innate immune surveillance, linking metabolic regulation of angiogenesis to immune evasion [159, 160].

#### 5.2.2 Macrophages

Macrophages represent another major innate immune population shaped by metabolic regulation. Within the tumor immune microenvironment, FBP1 is highly expressed in TAMs, where elevated FBP1 levels suppress glycolysis and favor polarization toward the immunosuppressive M2 phenotype. M2‐polarized TAMs secrete anti‐inflammatory cytokines and pro‐tumorigenic factors, thereby dampening antitumor immunity and supporting tumor growth. This cell‐intrinsic role of FBP1 in macrophages contrasts with its tumor‐suppressive role in cancer cells, illustrating the functional plasticity of this metabolic enzyme across different cell types. Large‐scale analyses in malignant gliomas reveal that high FBP1 expression correlates with increased infiltration of M2‐like TAMs and elevated immunosuppressive cytokines, including IL‐10 and TGF‐β1 [9]. Conversely, metabolic interventions such as short‐term fasting can reprogram TAMs and enhance the efficacy of anti‐PD‐L1 therapy in HCC models [10].

Notably, metabolic regulation of macrophage polarization is closely intertwined with inflammatory signaling and therapy resistance. Context‐dependent metabolic rewiring can reinforce immunosuppressive macrophage states that promote tumor progression and limit therapeutic efficacy, underscoring the importance of targeting macrophage metabolism as part of combination immunotherapy strategies [35, 73, 109, 123].

### 5.3 Metabolic Checkpoints Governing T Cell Function and Exhaustion

T cell‐mediated antitumor immunity is tightly constrained by metabolic checkpoints that regulate energy utilization, differentiation, and effector function within the TME. Dysregulated metabolic signaling can impose functional exhaustion on T cells, characterized by impaired proliferation, reduced cytokine production, and increased expression of inhibitory receptors. Emerging evidence suggests that metabolic enzymes act as intrinsic regulators of these checkpoints, linking cellular metabolism directly to T cell fate decisions. FBP1 serves as a pivotal metabolic checkpoint in T cells, where its dynamic expression integrates extrinsic signals from the TME to control T cell activation and exhaustion.

Under physiological conditions, FBP1 expression in T cells is relatively low, permitting active glycolysis to support proliferation and effector responses. However, under specific microenvironmental cues such as metabolic competition, chronic inflammation, or hormonal signaling, FBP1 expression can be markedly upregulated. Vitamin D‐activated vitamin D receptor (VDR) signaling has been shown to directly enhance FBP1 transcription in T cells, particularly γδ T cells, thereby suppressing glycolysis through inhibition of the Akt/p38 MAPK pathway and reducing the secretion of proinflammatory cytokines such as IL‐17 [146]. This metabolic restriction limits T cell inflammatory activity and effector capacity, demonstrating that upregulation of FBP1 acts as a cell‐intrinsic brake on T cell function.

In parallel, metabolic alterations in tumor cells indirectly impose metabolic stress on T cells. Loss of FBP1 in tumor cells leads to sustained activation of the STAT3 pathway and upregulation of PD‐L1, reinforcing immune checkpoint signaling and suppressing cytotoxic T cell function [5]. Moreover, enhanced glycolysis in FBP1‐deficient tumor cells exacerbates glucose competition and lactate accumulation, further constraining T cell metabolism and promoting functional exhaustion. Such metabolic constraints are further exacerbated by tumor cell‐intrinsic metabolic programs that enhance glycolysis, angiogenesis, and invasive growth, collectively intensifying nutrient competition and reinforcing T cell dysfunction within the TME [37, 147, 161, 162]. Clinical analyses indicate that tumors with high FBP1 expression, such as glioblastoma, exhibit reduced infiltration of functional T cells. These tumors are characterized by increased expression of exhaustion markers, including PD‐1 and CTLA‐4, as well as elevated immunosuppressive cytokines such as TGF‐β1 and IL‐10 [9].

Together, these findings identify FBP1‐mediated metabolic regulation as a central checkpoint governing T cell functionality and exhaustion. Modulating metabolic enzymes or pathways that restrict T cell metabolism may relieve exhaustion, restore effector activity, and enhance the efficacy of immune checkpoint blockade therapies.

### 5.4 Metabolism‐Driven Remodeling of Stromal and Fibrotic Tumor Niches

Beyond immune cells, tumor‐associated metabolic reprogramming profoundly reshapes stromal and fibrotic niches, which are increasingly recognized as active regulators of tumor progression and immune suppression. Stromal cells, particularly hepatic stellate cells (HSCs), respond dynamically to metabolic alterations in parenchymal cells, linking metabolic dysregulation to fibrosis‐driven tumor evolution. These stromal changes not only remodel tissue architecture but also generate a pro‐tumorigenic and immunosuppressive microenvironment. FBP1 loss acts as a critical initiator of this process, triggering metabolic and inflammatory cascades that drive stromal activation and fibrotic remodeling.

HSCs are the principal effector cells driving liver fibrosis and play a pivotal role in the transition from chronic liver injury to HCC [138, 139, 140]. Under physiological conditions, quiescent HSCs maintain extracellular matrix homeostasis and metabolic balance [141]. However, metabolic stress induced by chronic injury or oncogenic transformation promotes HSC activation and transdifferentiation into proliferative, extracellular matrix–producing myofibroblasts, thereby facilitating fibrosis and tumor progression [144, 145]. Liver‐specific deletion of FBP1 has been shown to disrupt glucose and fructose metabolism, triggering metabolic reprogramming that accelerates HSC activation and premature senescence, accompanied by increased secretion of inflammatory and profibrotic mediators [46]. This establishes FBP1 as a key metabolic gatekeeper whose loss directly licenses a pro‐tumorigenic stromal response.

Beyond HMGB1, related high‐mobility group proteins have also been implicated in metabolic‐inflammatory coupling within tumors. For instance, HMGB2 has been reported to promote tumor progression through metabolic enzyme activation and suppression of metabolic restraints, further highlighting the broader relevance of damage‐associated molecular patterns in shaping metabolically driven stromal and immune niches [76].

In addition to direct metabolic effects, metabolic enzyme dysregulation can induce paracrine inflammatory signaling that further amplifies stromal remodeling. FBP1 deficiency promotes the nuclear release of high‐mobility group box 1 (HMGB1), which functions as a damage‐associated molecular pattern (DAMP) to aberrantly activate intrahepatic HSCs [28]. This HMGB1‐driven signaling cascade reinforces fibrotic niche formation and establishes a feed‐forward loop linking metabolic dysfunction, chronic inflammation, and tumor‐promoting stromal remodeling.

**FIGURE 5:**
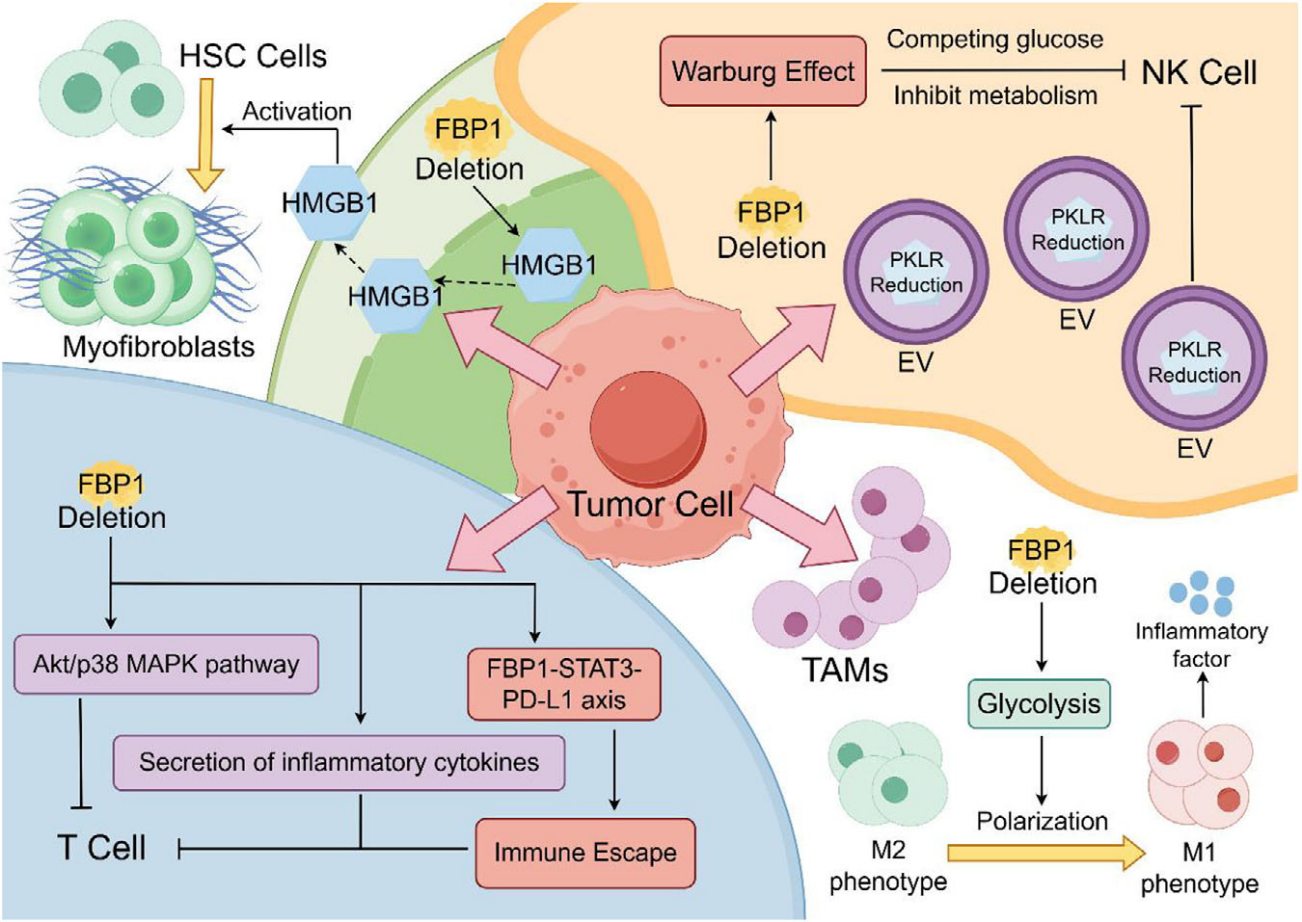
FBP1 deletion shapes the immunosuppressive TME by regulating a variety of immune cell functions. First, FBP1 deletion leads to enhanced glycolysis in tumor cells, competes with NK cells for glucose, and inhibits NK cell metabolism. PKLR is reduced in EVs secreted by tumor cells, which are taken up by NK cells and inhibit their activity. Second, FBP1 absence promotes glycolysis, M1 polarization, and inflammatory factor secretion in TAMs. In addition, tumor cells inhibit T cell effects by downregulating FBP1, promoting the Akt/p38 MAPK pathway, inflammatory factor secretion, and the FBP1‐STAT3‐PD‐L1 axis‐mediated immune escape. Finally, FBP1 deletion in hepatocytes triggers the release of HMGB1 from the nucleus, activating quiescent HSCs to transform into proliferating, extracellular matrix‐producing myofibroblasts, driving liver fibrosis and HCC progression. Akt, protein kinase B; EV, extracellular vesicle; HMGB1, high mobility group box 1; HSC, hepatic stellate cell; MAPK, mitogen‐activated protein kinase; NK cell, natural killer cell; PKLR, pyruvate kinase L/R; STAT3, signal transducer and activator of transcription 3; TAMs, tumor‐associated macrophages.

Overall, these findings highlight FBP1‐mediated metabolic dysregulation as a critical driver of stromal and fibrotic remodeling, a yet underappreciated dimension of tumor immunometabolism. Targeting metabolic pathways that govern stromal activation and fibrosis may disrupt the supportive tumor niche, restrain malignant progression, and enhance responsiveness to anticancer therapies. Consequently, the immunosuppressive TME is now understood not merely as a passive byproduct of tumor progression but as an active construct shaped by FBP1‐mediated metabolic reprogramming of diverse immune and stromal cells (Figure 5).

## 6 Targeting Immunometabolic Pathways: Therapeutic Strategies

Targeting immunometabolic pathways has emerged as a promising therapeutic strategy to restore immune function in cancer. Immune cells rely on tightly regulated metabolic programs to support activation, differentiation, and effector functions, while tumors exploit metabolic rewiring to induce immune suppression. Therapeutic interventions aimed at modulating glucose, lipid, amino acid, and mitochondrial metabolism can reprogram dysfunctional immune cells, alleviate metabolic competition within the TME, and enhance antitumor immunity. Importantly, immunometabolic targeting is increasingly recognized as a complementary approach to immune checkpoint blockade, with the potential to overcome resistance and improve treatment durability. Together, these strategies highlight metabolism not merely as a downstream consequence of immune activation, but as a druggable axis governing immune cell fate and therapeutic responsiveness.

As detailed in preceding sections, FBP1 serves as a prototypical immunometabolic hub that integrates tumor cell metabolism and immune checkpoint regulation. Its loss or dysregulation drives metabolic reprogramming, PD‐L1‐mediated immune evasion, and immunosuppression across multiple cancer types. Restoring FBP1 function—whether through pharmacological activation, gene‐based replacement, or modulation of upstream regulatory networks—therefore represents a compelling therapeutic paradigm that directly targets the intersection of metabolism and immunity. Building upon this conceptual framework, the following sections systematically review current strategies for immunometabolic intervention, spanning small‐molecule modulation, gene‐ and vitamin‐based approaches, nanotechnology platforms, and the emerging translational evidence base that supports their clinical application.

### 6.1 Targeting Metabolic Pathways to Modulate Immune Responses

Pharmacological modulation of metabolic pathways represents a promising strategy to reshape tumor–immune interactions. Enhancing the activity of key metabolic enzymes involved in gluconeogenesis has been proposed as a means to counteract aberrant glycolytic reprogramming in cancer cells and restore immune‐permissive metabolic states. To date, no highly selective and potent direct activators have been clinically validated; however, several small‐molecule agents have been reported to exert indirect activating effects. Inhibition of upstream signaling pathways, such as AKT or NRF2, may stabilize enzyme expression or activity by preventing proteasomal degradation, thereby partially restoring metabolic control [35].

Earlier biochemical studies demonstrated that magnesium ions function as essential cofactors that enhance enzymatic activity, highlighting the feasibility of metabolic fine‐tuning at the catalytic level [163]. Epigenetic modulation also provides an alternative approach: DNA demethylating agents, including 5‐azacytidine (5‐Aza‐CdR), have been shown to reactivate gene expression through promoter demethylation and suppress leukemia cell proliferation [164]. In addition, bioactive compounds derived from traditional Chinese medicine exhibit regulatory potential. β‐Elemene has been reported to induce enzyme expression and increase the sensitivity of lung cancer cells to gefitinib treatment [61], while Huai'er granules restore expression by suppressing the transcription factor Twist1 and are clinically applied in cholangiocarcinoma management [165].

Despite these advances, the development of direct metabolic activators remains at an early stage. Current evidence is largely based on indirect mechanisms or preclinical observations, and robust validation in vitro and in vivo is still required. Future efforts should integrate chemical screening, structural biology, and functional assays, with particular emphasis on specificity and safety, to minimize off‐target effects on related metabolic enzymes and ensure translational feasibility.

### 6.2 Gene‐, Vitamin‐, and Nanotechnology‐Based Metabolic Interventions

Beyond small‐molecule modulation, gene‐, vitamin‐, and nanotechnology‐based interventions provide complementary strategies to reprogram tumor immunometabolism at multiple regulatory layers. These approaches aim to restore metabolic balance, reshape the immunosuppressive TME, and enhance responsiveness to immunotherapy through transcriptional, epigenetic, and posttranscriptional mechanisms.

Vitamin‐mediated metabolic regulation has attracted increasing attention due to its safety profile and pleiotropic biological effects. Vitamin D and its active metabolites have been shown to transcriptionally upregulate metabolic enzymes by directly engaging promoter regions, thereby suppressing glycolytic flux and inhibiting tumor progression [146, 166, 167]. Vitamin D3 has also been reported to promote monocyte differentiation and macrophage maturation through metabolic reprogramming [168], while markedly restraining acute myeloid leukemia development by suppressing glycolysis [122]. Similarly, vitamin A derivatives, including retinoic acid, can enhance metabolic enzyme expression via receptor‐mediated transcriptional activation, leading to reduced glycolysis, antiangiogenic effects, and inhibition of tumor growth [161, 167, 169]. Vitamin C further contributes to metabolic reprogramming by activating TET2‐dependent DNA demethylation, restoring gene expression, and suppressing hypoxia‐driven signaling in renal cell carcinoma [87].

Gene‐based therapeutic strategies, particularly mRNA delivery, offer a direct means to restore metabolic enzyme expression in tumors characterized by metabolic dysregulation. mRNA‐based therapeutics allow transient, controllable protein expression without genomic integration, reducing the risk of insertional mutagenesis [170]. Advances in lipid nanoparticle (LNP) delivery systems have significantly improved the stability, targeting efficiency, and translational feasibility of mRNA therapeutics [171]. Preclinical studies demonstrate that nanoparticle‐encapsulated mRNA delivery can effectively restore metabolic enzyme function in tumor tissues, leading to reduced tumor growth and enhanced antitumor immune responses in vivo [62].

Nanotechnology further expands the therapeutic landscape by enabling precise metabolic and immunological modulation. Engineered nanomaterials facilitate targeted delivery of metabolic regulators, nucleic acids, and immunomodulatory agents, thereby overcoming key limitations of conventional therapies such as poor specificity, short half‐life, and systemic toxicity [172, 173]. In recent years, nanotechnology‐based approaches—including targeted therapy, photodynamic therapy (PDT) [174, 175], chemotherapy [176, 177, 178, 179, 180], molecular therapy [181, 182, 183, 184], and immunotherapy [185, 186, 187, 188, 189, 190]—have increasingly been integrated into cancer treatment paradigms by exploiting the unique physicochemical properties of nanomaterials, such as high surface‐to‐volume ratios, tunable optical absorption, superparamagnetic behavior, and favorable electrical characteristics, thereby enabling enhanced delivery precision, therapeutic efficacy, and immunomodulatory capacity [191].

Thus, these gene‐, vitamin‐, and nanotechnology‐based interventions represent versatile and scalable platforms for integrating metabolic regulation into precision cancer immunotherapy. Understanding the diverse mechanisms of FBP1 upregulation provides a foundation for developing targeted interventions that restore its tumor‐suppressive functions, recalibrate immunometabolic homeostasis, and enhance therapeutic efficacy in cancer (Table 3).

**TABLE 3:** Mechanisms of FBP1 upregulation.

Regulatory pathway	Regulatory factor	Mechanism of upregulation	References
FBP1 activators	AKT inhibitor/NRF2 inhibitor	Block degradation pathways of FBP1	[31]
	Magnesium ions	Enhance the catalytic activity of FBP1	[91]
	5‐Aza‐CdR	Promote promoter demethylation, restore FBP1 expression	[92]
	β‐elemene	Stimulate increased FBP1 expression	[36]
	Huaier granule	Inhibit Twist1 expression, restore FBP1 expression	[95]
Vitamins	VD	Promote FBP1 expression	[83, 96, 97, 98]
	VA	Promote FBP1 expression	[98, 100, 160]
	VC	Activate TET2 and promote FBP1 expression	[61]
mRNA‐based Therapy	FBP1 mRNA	Promote FBP1 translation	[59, 102]
HIF‐1α inhibitor	TET2	Decrease cancer cell dependence on glycolysis and promote FBP1 expression	[61]

Abbreviations: 5‐Aza‐CdR, 5‐aza‐2′‐deoxycytidine; AKT, protein kinase B; HIF‐1α, hypoxia‐inducible factor 1‐α; mRNA, messenger RNA; NRF2, nuclear factor erythroid 2‐related factor 2; TET2, tet methylcytosine dioxygenase 2; Twist1, twist family bHLH transcription factor 1; VA, vitamin A; VC, vitamin C; VD, vitamin D.

**FIGURE 6:**
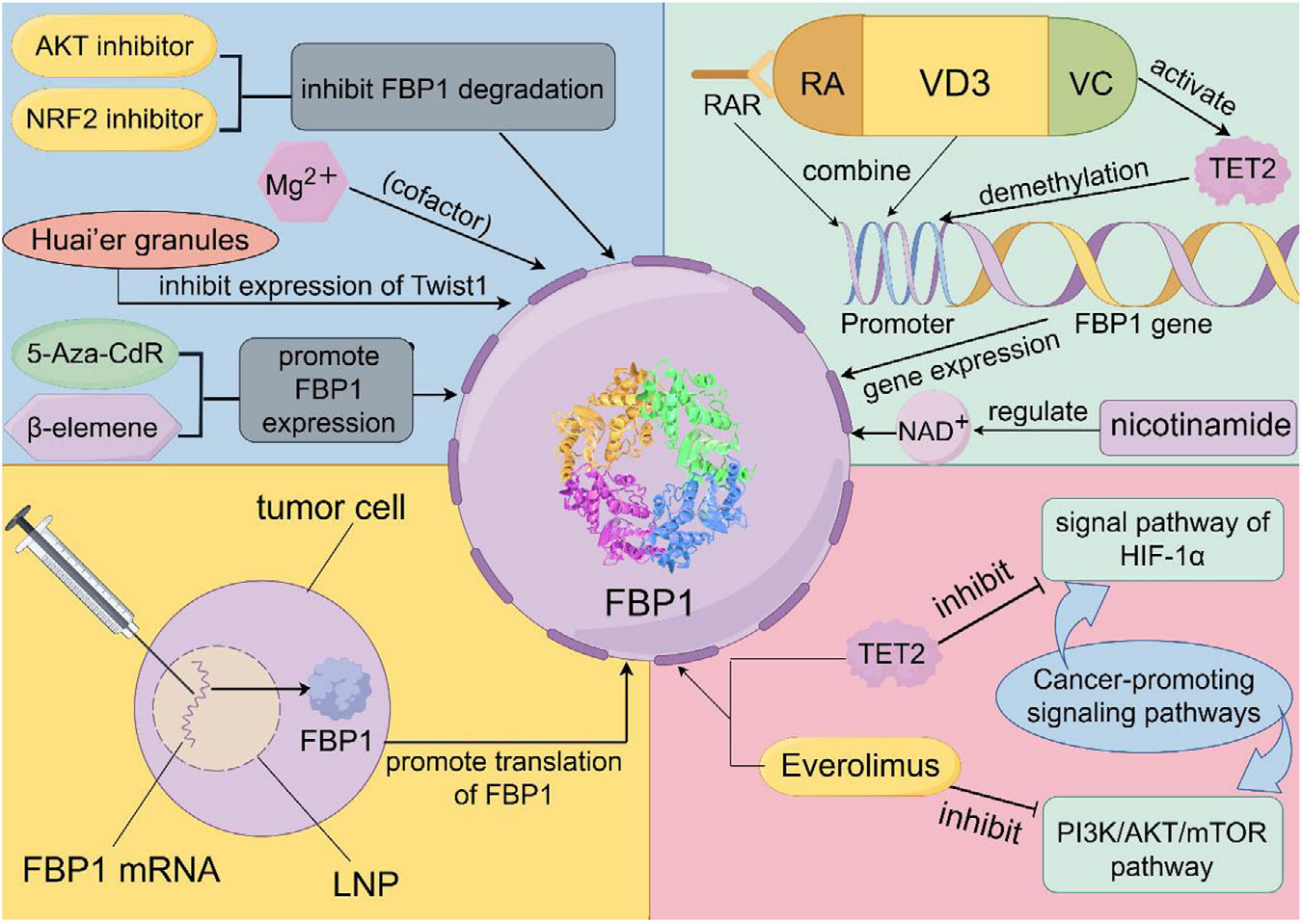
Upregulating the mechanism of cancer treatment of FBP1. AKT inhibitors and NRF2 inhibitors increase FBP1 levels by blocking FBP1 degradation, while magnesium ions can act as cofactors to activate FBP1 enzymatic activity. In addition, at the gene expression level, 5‐Aza‐CdR and β‐elemene can directly induce FBP1 expression, while Huai'er granules indirectly upregulate FBP1 levels by inhibiting the expression of Twist1. For example, RA, as a metabolite of VA, upregulates FBP1 expression on the FBP1 promoter through RAR binding; VD3 can act on the FBP1 promoter to promote its expression; VC promotes promoter demethylation by activating TET2, thereby enhancing FBP1 expression. In addition, niacinamide may indirectly affect FBP1 activity by modulating NAD^+^ levels. FBP1 mRNA was encapsulated in LNPs and delivered to tumor cells to restore FBP1 expression. From the perspective of signaling pathways, TET2 and Everolimus improved the functional environment of FBP1 by inhibiting the HIF‐1α signaling pathway and PI3K/AKT/mTOR pathway, respectively. OXPHOS, oxidative phosphorylation; TME, tumor microenvironment; STAT3, signal transducer and activator of transcription 3; TAMs, tumor‐associated macrophages; IL‐10, interleukin‐10; TGF‐β, transforming growth factor‐β; NRF2, nuclear factor erythroid 2‐related factor 2; VD3, vitamin D3; RA, retinoic acid; VC, vitamin C; TET2, ten‐eleven translocation 2; NAD+, nicotinamide adenine dinucleotide; LNP, lipid nanoparticle; mRNA, messenger RNA; HIF‐1α, hypoxia‐inducible factor‐1α; PI3K, phosphoinositide 3‐kinase; mTOR, mammalian target of rapamycin; 5‐Aza‐CdR, 5‐aza‐2'‐deoxycytidine.

Consequently, diverse interventional strategies—encompassing pharmacological inhibitors, vitamin‐mediated transcription, mRNA delivery, and nanotechnology—collectively demonstrate the therapeutic potential of restoring FBP1 function to recalibrate immunometabolic homeostasis and enhance antitumor efficacy (Figure 6).

### 6.3 Preclinical and Clinical Evidence for Immunometabolic Targeting: Pathway‐ and Mechanism‐Based Perspectives

Accumulating evidence indicates that immunometabolic regulation is a fundamental determinant of immune cell function and disease progression. Immune responses are tightly coupled to metabolic programs, including glycolysis, mitochondrial oxidative phosphorylation, amino acid metabolism, and metabolite‐mediated signaling. The concept of immunometabolism has established metabolic reprogramming as a foundational layer of immune regulation, providing a mechanistic framework for therapeutic intervention [192, 193].

#### 6.3.1 Molecular Mechanisms of Immunometabolism‐Driven Immune Suppression and Preclinical Evidence

Preclinical studies have demonstrated that T‐cell effector function is highly dependent on glucose availability and glycolytic flux. Glucose restriction markedly suppresses IFN‐γ production and cytotoxic activity in CD8^+^ T cells, whereas TCR/CD28 co‐stimulation enhances glucose uptake and glycolysis to support T‐cell activation [194]. In tumor models, metabolic competition represents a major immune evasion mechanism. Tumor cells increase glycolysis and lactate production, thereby reshaping the tumor immune microenvironment and suppressing infiltrating immune cells [195, 196]. Lactate dehydrogenase A (LDHA) plays a central role in tumor‐immune metabolic crosstalk. LDHA‐driven lactate accumulation promotes immunosuppressive cell expansion and impairs antitumor T‐cell function [197, 198]. Targeting this axis has shown efficacy in animal models: the LDHA inhibitor ML‐05 reduced intratumoral lactate, inhibited tumor growth, and restored Th1 and granzyme B^+^ CD8^+^ T‐cell‐mediated immunity, with further enhancement when combined with anti‐PD‐1 therapy or STING agonists [199]. Beyond glycolysis, dysregulated cholesterol metabolism contributes to immune suppression, and targeting cholesterol pathways has been shown to reprogram the immune microenvironment and enhance antitumor immunity [200]. Dysregulated metabolic pathways can establish an immunosuppressive microenvironment by impairing the cytotoxic function of innate and adaptive immune cells (Table 4).

**TABLE 4:** Metabolic mechanisms underlying immune suppression.

Metabolic pathway	Main experimental models	Mechanism
Lactate metabolism circRNA regulation [201]	HCC cell–NK cell co‐culture and NCG mouse orthotopic liver cancer model	circSMPD4 stabilizes LDHA, promotes lactate accumulation, and suppresses NK‐cell cytotoxicity and IFN‐γ production
Aerobic glycolysis (Warburg effect) [194]	Activated CD4^+^ T cells, Listeria monocytogenes–infected mice, and tumor–T cell co‐culture	GAPDH acts as a metabolic checkpoint controlling IFN‐γ mRNA translation
Glycolysis LDHA–lactate axis [197]	GBM mouse models (CT2A, GL261) and PDX	;lactate‐LDHA activates ERKYAP1–STAT3 signaling and establishes macrophage‐mediated positive feedback via EVs
Lactate metabolism LDHA enzymatic activity [199]	B16F10 melanoma syngeneic mouse model	LDHA inhibition reduces lactate levels and enhances Th1 and Granzyme B^+^ CD8^+^ T‐cell infiltration
Glycolysis BAP1–LDHA interaction [198]	Uveal melanoma cell lines and B16F10 tumor‐bearing mice	BAP1 loss causes LDHA accumulation, increased glycolysis, and lactate secretion
Glycolysis ATP production [202]	HeLa, HCT‐116 and MCF‐7 cell lines	Diclofenac inhibits LDHA, decreases ATP, and induces AMPK‐mediated metabolic stress

Abbreviations: AMPK, AMP‐activated protein kinase; ATP, adenosine triphosphate; BAP1, BRCA1‐associated protein 1; CD, cluster of differentiation; circRNA, circular RNA; ERK, extracellular signal‐regulated kinase; EVs, extracellular vesicles; GAPDH, glyceraldehyde‐3‐phosphate dehydrogenase; GBM, glioblastoma multiforme; HCC, hepatocellular carcinoma; IFN‐γ, interferon‐gamma; LDHA, lactate dehydrogenase A; mRNA, messenger RNA; NCG, NOD‐Prkdcem26Il2rgem26Nju (severely immunodeficient mouse); NK, natural killer; PDX, patient‐derived xenograft; STAT3, signal transducer and activator of transcription 3; Th1, T helper type 1; YAP1, Yes‐associated protein 1.

#### 6.3.2 Translational and Clinical Evidence Supporting Immunometabolic Targeting Strategies

Clinical and translational studies further support the relevance of immunometabolic dysregulation in human disease. Enhanced glycolysis, lactate accumulation, and lipid metabolic abnormalities in tumor tissues are consistently associated with immune exhaustion, immunosuppressive phenotypes, and poor clinical outcomes [192, 196]. Metabolites such as lactate act as context‐dependent immunomodulators linking metabolic state to immune function and have emerged as potential biomarkers for immunotherapy response [197, 202]. Glutamine metabolism represents another critical axis, as glutamine supports both tumor proliferation and immune cell differentiation. Modulation of glutamine metabolism has therefore been proposed as a strategy to enhance antitumor immunity and improve immunotherapy efficacy [203]. Based on these insights, immunometabolic targeting is increasingly explored in clinical settings. Early studies combining metabolic inhibitors with immune checkpoint inhibitors (ICIs) suggest enhanced efficacy in selected tumor types and patient subgroups [192, 199]. In stage III melanoma, the NADINA and SWOG‐1801 trials demonstrated superior outcomes with neoadjuvant versus adjuvant immune checkpoint blockade. In the Morpheus‐Melanoma trial (NCT05116202), the PD‐1/LAG‐3 bispecific antibody tobemstomig achieved a pathological response rate comparable to nivolumab plus ipilimumab, with a lower incidence of grade ≥3 adverse events. Biomarkers, including CD8^+^/CD3^+^ T‐cell infiltration, IFN‐γ signaling, tumor mutational burden, and preoperative circulating tumor DNA, were associated with treatment response [204]. Retrospective analyses further showed that high peak‐dose corticosteroid use for irAE management was associated with worse survival, underscoring the need to minimize peak immunosuppression during immunotherapy [205]. Emerging strategies, including JAK inhibition, metabolic risk stratification, and nanomedicine‐based drug delivery systems, further expand the scope of immunometabolic intervention by modulating immunosuppressive cell populations, improving therapeutic targeting, and reducing toxicity [206, 207, 208]. Nonetheless, challenges remain, including limited clinical trial sizes, metabolic pathway pleiotropy, and the lack of standardized immunometabolic biomarkers. Future large‐scale, multicenter studies integrating metabolomics and single‐cell approaches will be essential to optimize patient selection and combination strategies, facilitating the clinical translation of immunometabolic targeting. Therapeutic strategies targeting immunometabolic pathways can synergistically enhance immunotherapy efficacy by reprogramming the immunosuppressive TME, overcoming resistance to immune checkpoint inhibitors, and enabling biomarker‐guided patient stratification (Table 5).

**TABLE 5:** Translational and clinical evidence for metabolism‐targeted combination immunotherapy.

Metabolic pathway	Combination strategy rationale	Key mechanistic conclusions and clinical implications	Representative clinical/translational evidence and supporting data
Glycolysis [209]	Anti‐CSF‐1R + anti‐PD‐1 reprograms M2‐like TAMs to reverse their boost of tumor glycolysis, relieving metabolic suppression of effector T cells and overcoming ICI resistance	M2‐TAMs increase hypoxia and glycolysis, inhibiting glycolytically demanding CD4+/CD8+ effector T cells and expanding lipid‐oxidation‐dependent Tregs. CSF‐1R blockade repolarizes TAMs to M1, increases CD8+ T‐cell infiltration, and reverses this metabolic immune suppression	In mouse models, anti‐CSF‐1R + anti‐PD‐1 mAbs achieved total tumor eradication and a considerable survival benefit
Glutamine metabolism [203]	Block tumor glutamine addiction with GLS/uptake inhibitors (CB‐839, JHU083, V‐9302) combined with ICIs, chemo, or vaccines to boost antitumor immunity	JHU083 blocks tumor metabolism but enhances CD8+ T‐cell memory; CB‐839 synergizes with anti‐PD‐L1; glutamine antagonism reduces MDSCs/Tregs and increases Th1/CD8+ infiltration	CB‐839+everolimus: PFS benefit in RCC; +azacitidine: 62.5% mCR/HI in MDS. JHU083+anti‐PD‐1 effective in KEAP1‐mutant lung cancer. V‐9302+anti‐PD‐1 synergy in breast cancer
Lipid/cholesterol metabolism [201]	Anti‐PCSK9/ACAT1i/CYP27A1i + anti‐PD‐1/PD‐L1 to reverse cholesterol‐driven T‐cell dysfunction and boost CTLs	Context‐dependent: high cholesterol exhausts CD8+ T cells (ER stress/XBP1/PD‐1); low cholesterol impairs proliferation. ACAT1 inhibition potentiates CTLs; PCSK9 blockade enhances T‐cell infiltration; 27‐HC inhibition reduces immunosuppressive myeloid cells	Anti‐PCSK9+anti‐PD‐1 ↑ intratumoral CTLs. Avasimibe+anti‐PD‐1 synergistically suppressed tumors. CYP27A1i GW273297X+anti‐PD‐L1 improved efficacy and reduced metastasis
Metabolism‐driven ICI resistance [206]	JAKi + anti‐PD‐1 overcomes inflammatory ICI resistance by reversing T‐cell exhaustion and reshaping the TME	JAKi reduces MDSCs/Tregs, reverses chronic IFN‐driven exhaustion, and boosts CD8+ T‐cell proliferation; paradoxically, tempering chronic inflammation restores ICI responsiveness	Hodgkin: ruxo+nivo → 87% 2‑y OS (vs 23.8% ICI alone), ↓MDSCs, ↑CD8+. NSCLC: itacitinib+pembro → mPFS ∼2 y (vs. 6.5–10.3 mo), CD8+ burst, ↓exhaustion
Immune checkpoint cooperation (neoadjuvant) [204]	PD‐1/LAG‐3 bispecific tobemstomig targets Teff, not Treg, to reinvigorate TILs, overcome resistance, and improve benefit‐risk in neoadjuvant setting	Tobe matched nivo+ipi pRR (80% vs. 77%) with far less ≥3 TRAE (2.5% vs. 23%); adding TIGIT ineffective. High CD8, IFNγ, TMB, and ctDNA clearance predict response. Dual PD‐1/LAG‐3 safe and effective	Morpheus‐melanoma (NCT05116202): tobe (*n* = 40) pRR 80%, MPR 62.5%, ≥3 TRAE 2.5%; nivo+ipi (*n* = 22) pRR 77%, MPR 73%, ≥3 TRAE 23%. 68% responders cleared ctDNA pre‑surgery; CD8/IFNγ predicted MPR (*p *< 0.05)

Abbreviations: CSF‐1R, 27‐HC, 27‐hydroxycholesterol; ACAT1, acetyl‐CoA acetyltransferase 1; colony‐stimulating factor 1 receptor; ctDNA, circulating tumor DNA; CTLs, cytotoxic T lymphocytes; CYP27A1, cytochrome P450 family 27 subfamily A member 1; ER, endoplasmic reticulum; GLS, glutaminase; HI, hematologic improvement; ICIs, immune checkpoint inhibitors; JAKi, Janus kinase inhibitor; LAG‐3, lymphocyte activation gene‐3; mAbs, monoclonal antibodies; MDS, myelodysplastic syndromes; MDSCs, myeloid‐derived suppressor cells; mPFS, median progression‐free survival; MPR, major pathological response; NSCLC, non–small cell lung cancer; OS, overall survival; PCSK9, proprotein convertase subtilisin/kexin type 9; PFS, progression‐free survival; pRR, pathological response rate; TAMs, tumor‐associated macrophages; Th1, T helper type 1; TILs, tumor‐infiltrating lymphocytes; TMB, tumor mutational burden; TRAE, treatment‐related adverse event; Tregs, regulatory T cells; XBP1, X‐box binding protein 1.

## 7 Conclusion and Future Perspectives

Aberrant activation of oncogenic signaling pathways, particularly PI3K/AKT/mTOR and HIF‐1α, is a recurrent feature of cancer‐associated metabolic dysregulation and constitutes a major obstacle to effective therapeutic intervention. Targeting key nodes within these pathways offers a rational means to indirectly restore metabolic homeostasis by re‐establishing regulatory constraints on downstream metabolic enzymes. Notably, mTOR inhibitors such as everolimus have been reported to upregulate FBP1 expression in selected cancer models, although the mechanistic basis and tumor‐type specificity of this effect remain incompletely defined [210]. In parallel, inhibition of HIF‐1α signaling reduces tumor reliance on aerobic glycolysis and may permit broader metabolic rebalancing through suppression of glycolysis‐associated transcriptional programs, potentially via upstream regulators such as TET2 [87].

The availability of clinically approved agents targeting these pathways, including mTOR inhibitors used in renal cell carcinoma [211], substantially lowers the translational barrier for incorporating pathway‐level metabolic modulation into immunometabolic therapeutic frameworks.

This review establishes FBP1 as a central metabolic gatekeeper and a multifaceted tumor suppressor across diverse malignancies. We synthesize compelling evidence demonstrating that FBP1 deficiency—frequently orchestrated by epigenetic silencing, posttranslational modifications, or transcriptional repression within the TME—acts as a pivotal driver of oncogenesis. Loss of FBP1 fuels the Warburg effect, facilitates epithelial–mesenchymal transition, promotes angiogenesis, enables immune evasion (notably via PD‐L1 upregulation), and contributes to therapy resistance. Importantly, FBP1 exerts tumor‐suppressive effects not only through its canonical antagonism of glycolysis but also via noncanonical mechanisms, including protein–protein interactions that inhibit HIF signaling, modulate NF‐κB activity, and regulate autophagy and senescence. A particularly unifying theme highlighted herein is the role of FBP1 as a key regulator of the immunosuppressive TME, shaping the function of NK cells, T cells, macrophages, and stromal populations while restraining immune checkpoint signaling.

Despite the strong therapeutic rationale for restoring FBP1 function, several critical challenges define future research directions. A central conceptual hurdle is the context‐dependent functional duality of FBP1. While predominantly acting as a tumor suppressor, FBP1 exhibits pro‐tumorigenic roles in specific genetic and therapeutic contexts, as exemplified by its divergent effects in prostate cancer and ovarian cancer [109, 110, 118, 119]. This “double‐edged sword” phenomenon [212] underscores the necessity of understanding how tumor type, molecular subtype, metabolic state, interacting partners, and posttranslational modification patterns determine FBP1 function. Our systematic integration of these seemingly contradictory findings emphasizes that indiscriminate FBP1 modulation may yield unintended consequences, highlighting the need for precision‐guided strategies informed by molecular and metabolic context.

From a translational perspective, restoring FBP1 function remains an attractive yet complex therapeutic goal. Emerging strategies, including epigenetic modulation, targeting ubiquitin–proteasome regulators, vitamin‐mediated transcriptional activation, mRNA delivery, and inhibition of upstream oncogenic signaling, have shown promise in preclinical models. Notably, small activating RNA (saRNA) technology offers a particularly compelling avenue for transcriptional upregulation of endogenous FBP1, with early clinical experience in related targets supporting its feasibility [213, 214, 215, 216]. Compared with indirect regulatory approaches or exogenous mRNA delivery, saRNA‐based activation may provide a more durable and physiological restoration of FBP1 expression while minimizing immunogenicity and degradation risks. Ultimately, the successful clinical translation of FBP1‐targeted strategies will likely require biomarker‐guided patient stratification and rational combination approaches integrating metabolic modulation with immune checkpoint blockade or conventional therapies. Thus, by critically integrating metabolic, immune, and translational dimensions, this review not only consolidates current knowledge but also delineates a forward‐looking roadmap to harness FBP1 modulation for durable and safe cancer immunotherapy.

## Author Contributions

C.W.L., Z.Q.L., and D.Z.K. wrote and edited the article. Z.Z.L., Y.M.Y. and Y.Y.D. visualized and investigated the article. L.L.Z. and J.N.C. reviewed the article. L.F.L. and G.S. supervised and conceptualized the article. C.W.L., Z.Q.L.,. and D.Z.K. made equal contributions to the manuscript. All authors read and approved the final manuscript.

## Funding

This work was supported by the National Science Foundation of China (Grant 32370976); the Collaborative Innovation Major Project of Zhengzhou (Grant No. 20XTZX08017); Funding for Scientific Research and Innovation Team of The First Affiliated Hospital of Zhengzhou University (Grant ZYCXTD2023005); Wu Jieping Medical Foundation Special Fund for Targeted Cancer Research (Grant 320.6750.2023‐02‐1); Henan Provincial Young Talent Support Project (Grant 2025HYTP085); Key Scientific Research Project in Henan Higher Education Institutions (Grant 26A360024); 2025 Discipline Development Project of The First Affiliated Hospital of Zhengzhou University (Grant 2025XKPY027); Discipline Development Project of The First Affiliated Hospital of Zhengzhou University(2025XKPY027);2026 Henan Provincial University Science and Technology Innovation Talent Support Program; 2026 Henan Provincial Natural Science Foundation Youth Science Fund Project (Category B);State Key Laboratory of Metabolic Dysregulation & Prevention and Treatment of Esophageal Cancer, Tianjian Laboratory of Advanced Biomedical Sciences, Zhengzhou University, Zhengzhou, China: 2025SGAQZ‐PY‐04; Key Research and development projects in Henan province(241111314000); Provincial Talent Program Grant (264000510005).

## Ethics Statement

This study did not involve human participants and/or animals or informed consent. Thus, ethical clearance is not applicable to this article.

## Conflicts of Interest

The authors declare no conflicts of interest.

## Data Availability

No data were used for the research described in the article.
